# Lipidomic analysis of moss species *Bryum pseudotriquetrum* and *Physcomitrium patens* under cold stress

**DOI:** 10.1002/pei3.10095

**Published:** 2022-12-22

**Authors:** Yi Lu, Finnur Freyr Eiriksson, Margrét Thorsteinsdóttir, Henrik Toft Simonsen

**Affiliations:** ^1^ Department of Biotechnology and Biomedicine Technical University of Denmark Kongens Lyngby Denmark; ^2^ ArcticMass Reykjavik Iceland; ^3^ Faculty of Pharmaceutical Sciences University of Iceland Reykjavik Iceland; ^4^ Laboratoire Biotechnologies Végétales plantes aromatiques et médicinales, Faculté des sciences Université Jean Monnet Saint‐Étienne Cédex 2 France

**Keywords:** biomarker, *Bryum pseudotriquetrum*, cold stress, lipidomics, *Physcomitrium patens*

## Abstract

Bryophytes, which lack lignin for protection, support themselves in harsh environments by producing various chemicals. In response to cold stress, lipids play a crucial role in cell adaptation and energy storage. Specifically, bryophytes survive at low temperatures by producing very long‐chain polyunsaturated fatty acids (vl‐PUFAs). The in‐depth understanding of the lipid response to cold stress of bryophytes was studied by performing lipid profiling using ultra‐high‐performance liquid chromatography‐quadrupole time of flight mass spectrometry (UHPLC‐QTOF‐MS). Two moss species (*Bryum pseudotriquetrum* and *Physcomitrium patens*) cultivated at 23°C and at 10°C were included in this study. Relative quantitative lipid concentrations were compared and the potential lipid biomarkers were identified by multivariate statistical analysis in each species. In *B. pseudotriquetrum*, it was observed that the phospholipids and glycolipids increased under cold stress, while storage lipids decreased. The accumulation of the lipids with high unsaturation degrees mostly appears in phospholipids and glycolipids for both mosses. The results also indicate that two unusual lipid classes in plants, sulfonolipids and phosphatidylmethanol are biosynthesized by the bryophytes. This has not been seen previously and show that bryophytes have a very diverse chemistry and substantially different from other plant groups.

## Graphical abstract

During cold stress hundreds of lipids were identified with high quality MS/MS spectrums in moss *Bryum pseudotriquetrum* and *Physcomitrium patens*. The lipidomes of *B. pseudotriquetrum* is reported for the first time, along with several unusual lipids.

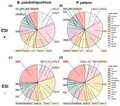

## 1 INTRODUCTION

Bryophytes, some of the smallest plants on the planet, have high chemical complexity and produce various metabolites, and naturally produce high amounts of very long‐chain polyunsaturated fatty acids (vl‐PUFAs, i.e., ≥20 carbons, ≥2 unsaturation degrees), including arachidonic acid (AA, 20:4) and eicosapentaenoic acid (EPA, 20:5; Girke et al., 1998; Lu et al., 2019). The presence of vl‐PUFAs is uncommon in vascular plants; however, it is known that vl‐PUFAs are essential for bryophytes to survive at low temperatures. Some bryophytes even exist in very harsh environment such as the Arctic and Antarctic since they are capable of survive during hard frost (Beike et al., 2014, 2015; Glime, 2017).

Low (<10°C) or freezing temperature usually leads to changes in the lipid composition and fatty acid desaturation in plants. The accumulation of vl‐PUFAs can lower the solidification point of the cells and keep the plant membrane fluidity together with other lipids (Tarazona et al., 2015). Although the lipid compositions of different moss species were reported before to contain high contents of vl‐PUFAs, most of the studies only examined their free fatty acid profiles using conventional lipid analysis method such as thin‐layer chromatography (TLC) or gas chromatography (GC; Dembitsky et al., 1993; Gellerman et al., 1975; Hartmann et al., 1986; Pejin et al., 2012). Recent development of lipidomics approaches allow a fast, high sensitivity and detailed molecular structural analysis of lipids. Herein, we performed plant lipidomics by ultra‐high‐performance liquid chromatography‐quadrupole time of flight mass spectrometry (UHPLC‐QTOF‐MS) of two moss species to achieve a broader coverage of the moss lipidomes.

In this study, we present the lipid composition in two moss species, and report the changes in lipid composition under cold stress when cultivated at 23 and 10°C by using a lipidomics approach. *Bryum pseudotriquetrum* was selected for its relative fast growth rate at both 23 and 10°C in liquid culture. Second, *Physcomitrium patens* was examined as a model organism for studying non‐seed plants (Rensing et al., 2020) and have been studied previously for it lipid composition (Girke et al., 1998). The genome of *P. patens* in contrast to higher plants has several enzymes involved in lipid metabolism such as Δ6‐desaturase, Δ5‐desaturase, and Δ6‐elongase for desaturation and elongation for synthesis of C18‐above fatty acids (Beike et al., 2014; Domergue et al., 2005). The lipidome of *P. patens* described previously consists of phospholipids including phosphatidylcholines (PC), phosphatidylethanolamines (PE), phosphatidylglycerols (PG), phosphatidylinositols (PI), and phosphatidic acid (PA), glycolipids including monogalactosyldiacylglycerols (MGDG), digalactosyldiacylglycerols (DGDG), and sulfoquinovosyldiacylglycerols (SQDG), storage lipids including diglyceride (DG) and triglyceride (TG), sphingolipids ceramides (Cer), free fatty acids (FA), and lysophosphalipids (LPL; Mikami & Hartmann, 2004; Resemann et al., 2021). Specifically, few studies confirmed that low temperature leads to the accumulation of the PUFAs in glycolipids (MGDG and DGDG) lipid class in bryophytes (Aro & Karunen, 1988; Beike et al., 2015). A very recent study in *P. patens* reported an increase in the levels of unsaturation of sphingolipids when exposing to cold temperature, and that mosses undergo a different response pathway than seed plants during low‐temperature acclimation (Resemann et al., 2021). The mechanism has not yet been confirmed in other moss species.

In this study, the lipid composition in different species, *B. pseudotriquetrum* and *P. patens*, were investigated and the lipid molecular changes during cold stress were examined. Additionally, potential lipid biomarkers that are upregulated and downregulated under cold stress were identified, and the relative concentrations of identified lipid molecular species and the total amount of each lipid class were quantified. The changes and the impact of these are discussed.

## 2 MATERIAL AND METHODS

### 2.1 Chemicals

HPLC‐grade chloroform and methanol, LC–MS‐grade acetonitrile, isopropanol, ammonium acetate, tributylamine, and potassium chloride (KCl, purity≥99.0%) were all purchased from Sigma‐Aldrich. Hexakis(2,2,3,3‐tetrafluoropropoxy)phosphazene was purchased from Apollo Scientific Ltd, UK. Glass tubes with PTFE coated caps was used for the analysis (DWK Life Sciences, UK). Ultra‐pure water was obtained from a Milli‐Q system.

### 2.2 In vitro cultivation

*P. patens* was obtained from the Moss Stock Center at the University of Freiburg, and sterilized *B. pseudotriquetrum* was obtained from Nils Cronberg, Lund University. *B. pseudotriquetrum*, together with four other Icelandic mosses *Racomitrium lanuginosum*, *Dicranella heteromalla, Rhytidiadelphus loreus, Pagonatum aloides*, and the model moss *P. patens* were preliminary tested for its in vitro growth ability. Visually *B. pseudotriquetrum* clearly showed highest growth rate at both 23 and 10°C of the six, and this species was chosen for evaluation of lipid profiling under cold stress together with the model species *P. patens*.

Moss liquid culture was grown in Knop media (Reski & Abel, 1985). Prior to the experiment, moss culture was blended in sterile water for 30 s and inoculation started with a biomass concentration of 100–300 mg/L. Two hundred milliliters of blended moss liquid culture was inoculated in 600‐ml cell culture flasks (Corning) and kept on a cell culture rocker, and two biological replicates were grown. In the standard condition, the moss liquid cultures were maintained in a growth chamber with a temperature of 23 ± 1°C, and the light intensity was kept at 15–20 Wm^−2^ as described by Pan et al. (2015). Cold‐stressed moss was kept in a growth chamber at 10 ± 1°C with the same light intensity as the standard condition. Day/night cycle was 16 h/8 h in both conditions. One hundred milliliters of biomass was harvested at day 21 from each biological replicate for extraction of moss at room temperature and the rest of the biomass was left at 10°C, and then harvested after another 24 h. The harvested biomass was filtered using Nylon cell strainers (pore size 70 μm) and kept at −20°C until extraction.

### 2.3 Internal standards

EQUISPLASH and DGTS d9 were purchased from Avanti Polar Lipids (Alabasterm). A 10 μl of 100 μg/ml solution containing each internal standard (IS) was spiked to the samples. The ISs include phosphatidylcholine (PC) 15:0–18:1(d7), phosphatidylethanolamine (PE) 15:0–18:1(d7), phosphatidylglycerol (PG) 15:0–18:1(d7), phosphatidylinositol (PI) 15:0–18:1(d7), phosphatidylserine (PS) 15:0–18:1(d7), lysophosphatidylcholine (LPC) 18:1(d7), lysophosphatidylethanolamine (LPE) 18:1(d7), diglyceride (DG) 15:0–18:1(d7), triglyceride (TG) 15:0–18:1(d7)‐15:0, MG 18:1(d7), cholesterol ester (CE) 18:1(d7), sphingomyelin (SM) d18:1/18:1(d9), sphingolipids ceramide (Cer) 18:1;2O/16:0(d7), and diacylglyceryl‐N,N,N‐trimethylhomoserine (DGTS) d9.

As isotope‐labeled lipid internal standards are not all commercially available, Welti et al. (2002) used hydrogenated MGDG and DGDG as internal standards for plant lipid profiling since those lipids do not exist in *Arabidopsis thaliana*. However, hydrogenated glycolipids are not suitable for using as internal standards for bryophytes because we have detected DGDG 32:0 (16:0_16:0) and SQDG 32:0 (16:0_16:0) produced by *P. patens* in our test run. Therefore, a deuterated glycolipid DGTS (d9) was used for glycolipids (MGDG, DGDG, and SQDG) quantification.

### 2.4 Lipid extraction

Due to the low growth rate of mosses, the harvested moss material in biological replicates were combined in each growth condition, and the combined material was divided into three aliquots for lipid extraction. The fresh moss material was first ground in liquid nitrogen. In each aliquot, 200 mg fresh frozen moss material was weighed into a glass tube; 3 ml chloroform/methanol (2:1, v/v) was added into the tube together with IS. The mixture was ultrasonicated for 20 min in dark, and then vortexed on a vortex mixer before adding 0.75 ml 1 M KCl. The mixture was vortexed again and centrifuged at 2000*g* for 5 min at 4°C. The organic phase was collected into a new glass tube by using a Pasteur pipette. The remaining mixture was washed twice with 1 ml chloroform, vortexed, and centrifuged; the organic phases were combined and evaporated under nitrogen stream and stored at −20°C until analysis. The dried lipid residue was re‐suspended in 150 μl of reconstitution solvent (one portion of chloroform/methanol (1:1, v/v) and nine portions of isopropanol/acetonitrile/water (2:1:1, v/v/v)). The solution was transferred to an Eppendorf tube and centrifuged at 13,000*g* for 5 min at room temperature, 100 μl of the supernatant was transferred to an HPLC vial with a glass insert for analysis. Quality control (QC) was prepared by pooling 10 μl aliquots of all samples.

### 2.5 Lipid quantification and recovery

To perform relative quantification of detected lipids, calibration curves of each IS were made with at least six concentration points (Table 1). Eight ISs were used for quantification in ESI+ mode and five in ESI‐ mode. The lowest quantitative concentration was 0.125 μg/ml and good linearity was observed up to 40 μg/ml. All ISs had a *R* ^2^ higher than .99.

**TABLE 1:** Internal standard used for calculation of lipid relative concentration

Compound	*m*/*z*	Adduct	Linear range (μg/ml)	Equation	*R* ^2^
PC 15:0_18:1(d7)	753.6134	[M + H]+	0.125–20	*y* = 278,979*x* − 27,702	.9966
LPC 18:1(d7)	529.3993	[M + H]+	0.125–20	*y* = 324,275*x* − 26,672	.9947
CE 18:1(d7)	675.6779	[M + NH_4_]+	5–40	*y* = 2465.4*x* − 7519.7	.9943
MG 18:1(d7)	381.3704	[M + NH_4_]+	1–40	*y* = 1582.3*x* + 240.42	.9994
DG 15:0_18:1(d7)	605.5845	[M + NH_4_]+	0.125–40	*y* = 71,212*x* + 3263.7	.9925
TG 15:0_18:1(d7)_15:0	829.7985	[M + NH_4_]+	0.125–40	*y* = 281,188*x* + 77,072	.9914
SM 18:1;2O/18:1(d9)	738.6470	[M + H]+	0.125–40	*y* = 159,238*x* + 9945.7	.9997
Cer 18:1;2O/15:0(d7)	531.5477	[M + H]+	0.125–20	*y* = 278,554*x* − 11,784	.9968
DGTS (d9)	721.6658	[M + H]+	0.125–2.5	*y* = 2 E+06*x* + 182,473	.995
PE 15:0_18:1(d7)	709.5519	[M − H]−	0.125–20	*y* = 126,515*x* + 800.14	.9985
PS 15:0_18:1(d7)	753.5418	[M − H]−	0.125–20	*y* = 57,483*x* + 12,403	.9971
PG 15:0_18:1(d7)	740.5466	[M − H]−	0.125–20	*y* = 213,728*x* + 44,408	.9969
PI 15:0_18:1(d7)	828.5626	[M−H]−	0.125–20	*y* = 162,279*x* + 45,604	.9917
LPE 18:1(d7)	485.3378	[M−H]−	0.125–20	*y* = 194,625*x* + 5032.5	.9961

Abbreviations: CE, cholesterol ester; Cer, ceramide; DG, diglyceride; DGTS, diacylglyceryl‐N,N,N‐trimethylhomoserine; LPC, lysophosphatidylcholine; LPE, lysophosphatidylethanolamine; MG, monodiacylglyceride; PC, phosphatidylcholine; PE, phosphatidylethanolamine; PG, phosphatidylglycerol; PI, phosphatidylinositol; PS, phosphatidylserine; SM, sphingomyelin; TG, triglyceride.

The recovery of the ISs was tested by comparing the peak areas of their corresponding m/z before (pre‐spike) and after (post‐spike) extraction procedure. For lipids that generate more than one form of ions, the most abundant ion form was chosen for relative quantification Most of the internal standards showed more than 85% recovery (Figure S1). PI 15:0–18:1(d7) had with the lowest recovery in both ESI+ and ESI‐ mode (73% and 78%, respectively), as expected (Aldana et al., 2020).

### 2.6 UHPLC‐QTOF‐MS analysis

UHPLC‐QTOF‐MS was performed on an Agilent Infinity 1290 UHPLC system (Agilent Technologies) coupled with Agilent 6545 QTOF MS with Dual Jet Stream ESI source. Samples were separated on an ACQUITY UPLC HSS T3 column (100 Å, 1.8 μm, 2.1 × 150 mm). The flow rate was 0.4 ml/min and the column temperature was 55°C. Solvent A consists of acetonitrile/H_2_O (60:40, v/v) and solvent B was isopropanol/acetonitrile (90:10, v/v), both supplied with 10 mM ammonium acetate. Linear gradient started from 40% solvent B and increased to 100% B in 10 min, and held at 100% B for 2 min, then reconditioned to 40% B in 2.5 min. Total analysis time was 15 min. The autosampler temperature was 8°C. Injection volume was 2 μl in positive ionization (ESI+) mode and 5 μl in negative ionization (ESI−) mode. Mass range was 100–1700 Da for MS scan and 30–1700 Da for MS/MS scan. Data were recorded in positive and negative ionization mode with an acquisition rate of 10 spectra/s in centroid profiles. Fragmentations were recorded with fixed collision energies of 10, 20 and 40 eV with maximum three precursors per cycle. Lock mass solution 1 μM tributylamine and 10 μM hexakis (2,2,3,3‐tetrafluoropropoxy)phosphazene with m/z 186.2216 and 922.0098 [M + H]^+^ in ESI+ mode and m/z 966.0012 [M + COOH]^−^ in ESI‐ mode.

### 2.7 Data analysis

Agilent MassHunter Qualitative Analysis (B.07.00) was used for firsthand chromatogram visualization and for integration of the internal standards. The raw data files were imported directly to MS‐DIAL (version 4.60) for further data analysis (peak peaking, deconvolution, compound identification, and alignment). Data normalization was achieved by calculating relative concentrations by using the internal standard representing the same lipid class. Parameters used for importing data is available at Data S1. The batch results used a set of criteria such as *m*/*z* error <0.01 Da compared to the theoretical mass, RSD < 30% of the replicates, and retention time deviation <0.05 min to ensure data quality (Broadhurst et al., 2018). The alignment result was imported to SIMCA 17 (Umetrics AB) for Principal Components Analysis (PCA) and Projection to Latent Structures with Discriminant Analysis (PLS‐DA; Bruce et al., 2008; Eriksson et al., 2006, 2008). Data were pareto‐scaled (to keep the original impact of the raw data) and log2‐transformed (to correct the skewed distributions; van den Berg et al., 2006). *T*‐tests were performed for calculating statistical significance of total lipid concentration changes between the moss species under different temperatures.

## 3 RESULTS

### 3.1 Lipid compositions in *B. pseudotriquetrum* and *P. patens*

**FIGURE 1:**
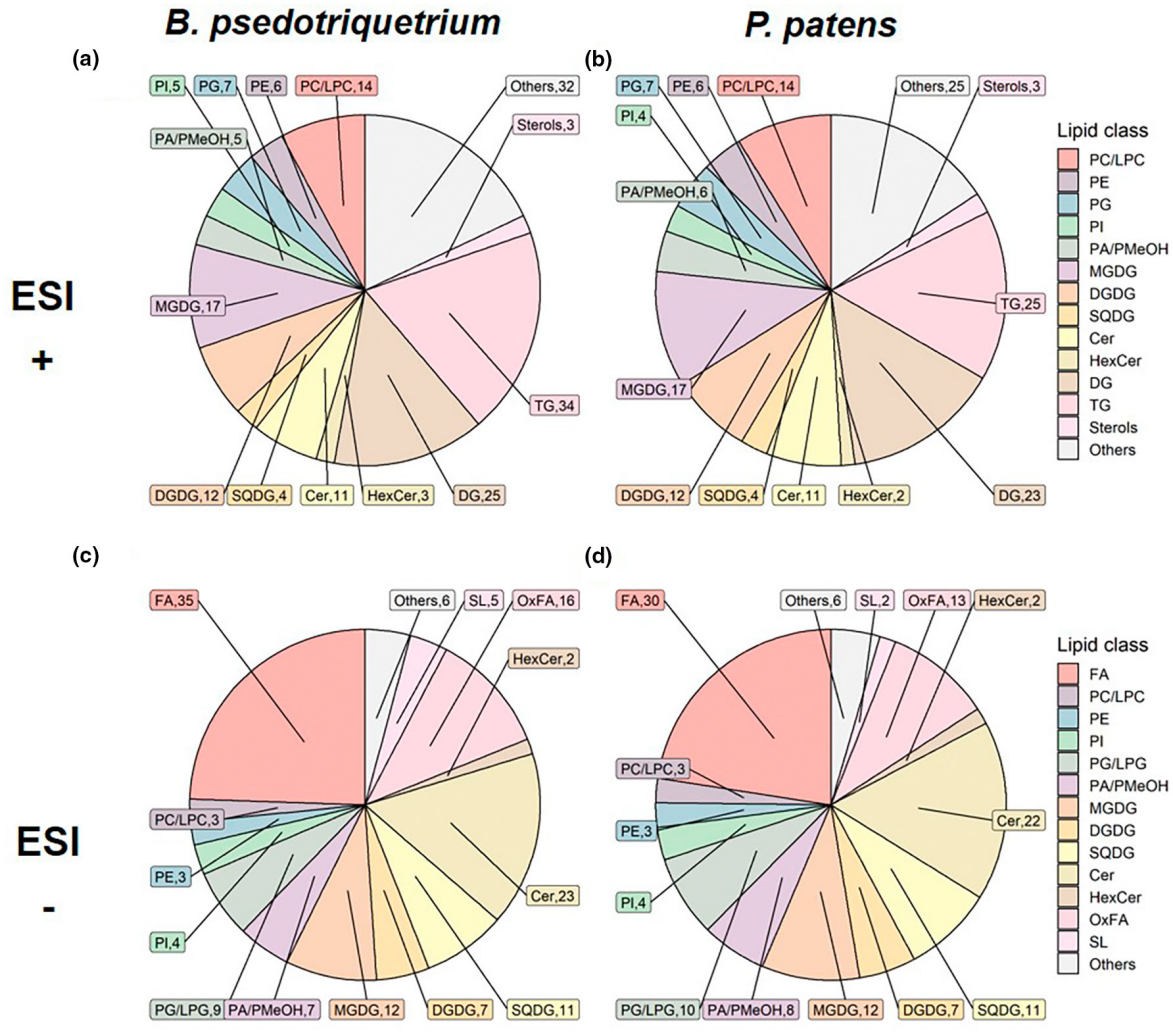
Detected lipid classes and the numbers of lipid molecular *species in* (a) *B. pseudotriquetrum in ESI+*, (b) *P. patens in ESI+*, (c) *B. pseudotriquetrum* in ESI‐, and (d) *P. patens* in ESI‐. Cer, ceramide; DG, diglyceride; DGDG, digalactosyldiacylglycerol; FA, fatty acid; HexCer, hexosylceramide; LPC, lysophosphatidylcholine; MGDG, monogalactosyldiacylglycerol; OxFA, oxidized fatty acid; PA, phosphatidic acid; PC, phosphatidylcholine; PE, phosphatidylethanolamine; PG, phosphatidylglycerol; PI, phosphatidylinositol; PMeOH, phosphatidylmethanol; SL, sulfonolipid; SQDG, sulfoquinovosyldiacylglycerol; TG, triglyceride

To review the lipid composition in *B. pseudotriquetrum* and *P. patens*, the identified lipids were classified accordingly to their lipid classes in ESI+ and ESI‐ mode, the lipid classes and the corresponding numbers of lipid metabolites are shown in Figure 1. In total, 204 features in ESI+ mode and 176 in ESI‐ mode were identified in the whole dataset with high‐quality MS2 spectra, including different adducts of the same lipid molecular species. After selecting the adduct with the most abundant intensity of a lipid molecular species, 178 lipid metabolites were detected in ESI+ mode and 143 in ESI‐ mode in *B. pseudotriquetrum*, whereas 159 lipid metabolites were detected in ESI+ mode and 133 in ESI‐ mode in *P. patens*. A detailed quantified list of identified lipid metabolites can be found in Data S1. Similar lipid metabolites could be found in both species, but the amount of each lipid metabolite varied (Data S3).

The major lipids found in both moss species are phospholipids such as PC, PE, PG, and PI, glycolipids MGDG, DGDG, and SQDG, signaling lipid Cer, and storage lipids DG and TG. Two unusual lipid classes phosphatidylmethanol (PMeOH) and sulfonolipids (SL) were detected in both moss species, along with considerable amounts of vl‐PUFAs detected in ESI‐ mode as free fatty acids (FA). The most abundant FA were 20:4 and 20:5 in *B. pseudotriquetrum*, and 16:0 in *P. patens* (Data S3). In addition, longer‐chain‐FAs with more than 20 carbons, such as saturated or mono‐saturated C22‐C25 FAs, were detected in both moss species.

### 3.2 Identification of biomarkers under cold stress

To identify potential lipid biomarkers from *B. pseudotriquetrum* and *P. patens*, chemometric approaches were applied by using unsupervised PCA and supervised PLS‐DA. First, PCA plots were generated to visualize the group information and to monitor the quality of the data (Figure S2). The two‐moss species show clear separation and the quality control samples are clustered tightly together, indicating that the batch is of good quality. *B. pseudotriquetrum* shows higher inter‐species variations between room temperature and cold temperature in ESI+ mode, this may indicate that *B. pseudotriquetrum* has a stronger response to cold stress than *P. patens*.

**FIGURE 2:**
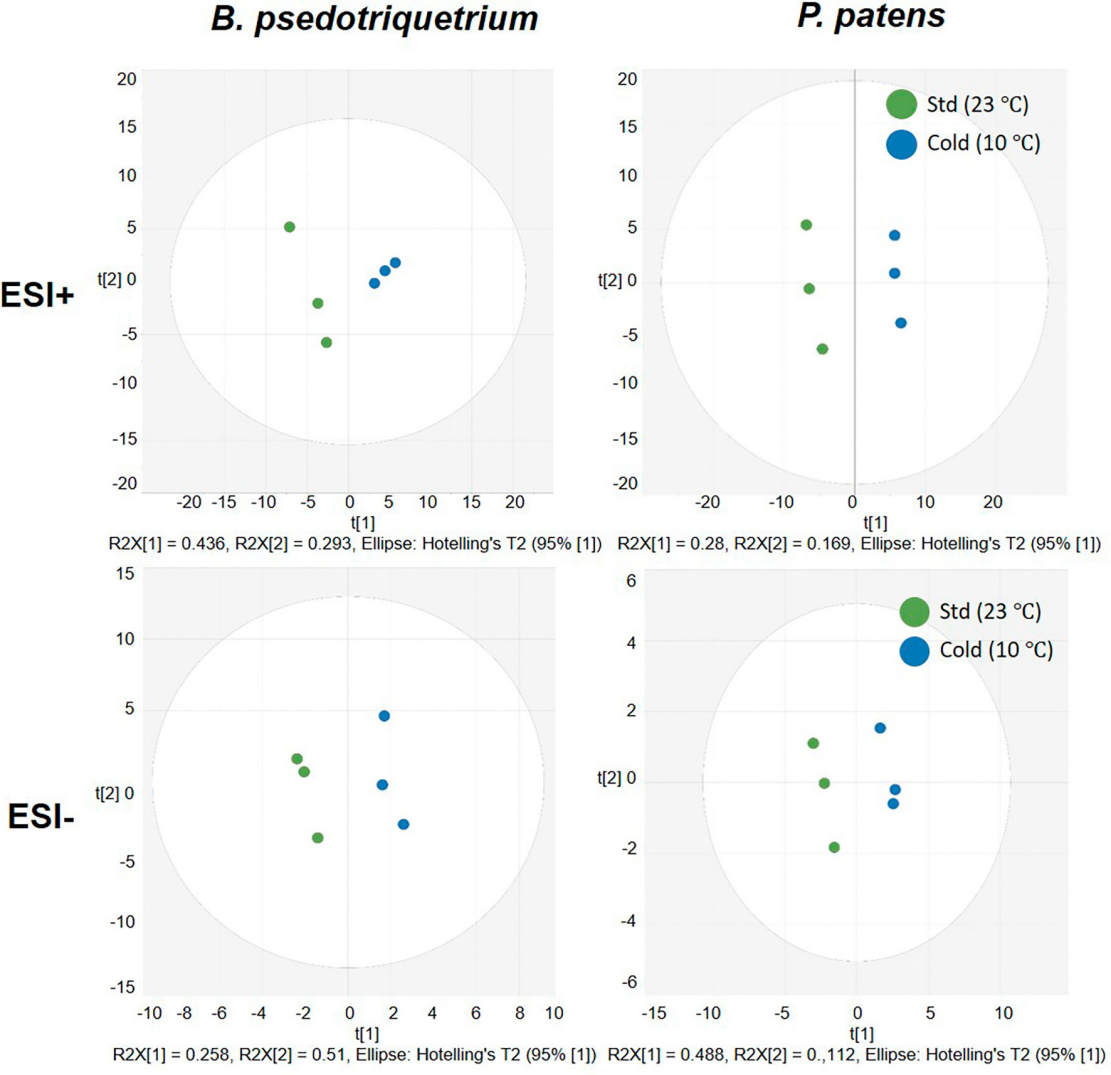
PLS‐DA score plot of *B. pseudotriquetrum* at room temperature and under cold stress in ESI+ mode (*R* ^2^ *X* = 0.449, *R* ^2^ *Y* = 0.997, *Q*2 = 0.676) and in ESI‐ mode (*R* ^2^ *X* = 0.728, *R* ^2^ *Y* = 0.961, *Q*2 = 0.804), *P. patens* at room temperature and under cold stress in ESI+ mode (*R* ^2^ *X* = 0.768, *R* ^2^ *Y* = 0.986, *Q*2 = 0.693) and in ESI‐ mode (*R* ^2^ *X* = 0.893, *R* ^2^ *Y* = 0.969, *Q*2 = 0.740)

To discriminate the samples that belong to 23 and 10°C in each species, individual PLS‐DA plots were built for *B. pseudotriquetrum* and *P. patens* for ESI+ and ESI‐ mode dataset, respectively (Figure 2). During this process it was also clear that an OPLS‐DA did not fit the data, possibly due to the small numbers of samples, thus PLS‐DA was chosen. All PLS‐DA plots showed clear separation of two groups of samples with high cumulative X and Y matrix variations (*R* ^2^ *X* and *R* ^2^ *Y*, respectively) and high predictability (The second quartile, Q2). As seen in Figure 2, the PLS‐DA plots resulted in one predictive and two orthogonal components. A total X variance (*R* ^2^ *X*) of 0.449 can be explained, and the predicted variance *R* ^2^ *Y* was 0.961. The predictive ability *Q*2*Y* = 0.676, which indicates good predictability (Eriksson et al., 2006). To test the validity of the PLS‐DA plots, permutation tests with 999 iterations were performed for all four PLS‐DA plots. The permutation tests are shown in Figure S2. Normally, a well‐fitted plots should have an intercept of *R* ^2^ smaller than 0.4 and *Q*2 should be smaller than 0.05, for lesser well fitted the slope of *R* ^2^ and *Q*2 should be larger than 0 (Bruce et al., 2008; Eriksson et al., 2008; Trygg et al., 2007). Here all of the *Q*2 intercepts were below 0 but the *R* ^2^ are greater than 0.4, and the slopes of both *R* ^2^ and *Q*2 were larger than 0, thus, the PLS‐DA plots can be considered to provide a good indication of the change in the lipid composition as a result of the cold stress.

**FIGURE 3:**
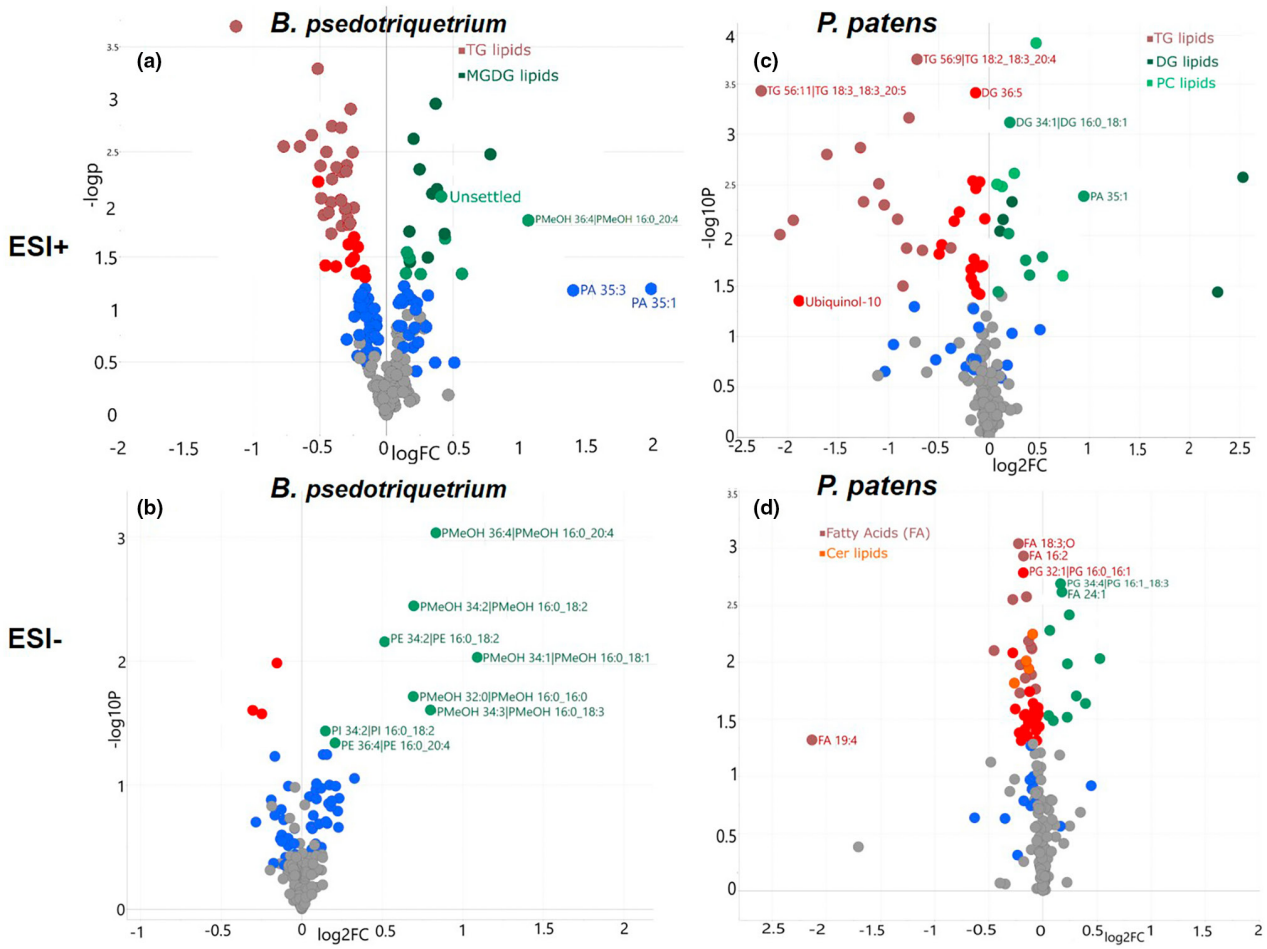
Volcano plots of significant changes of identified lipid metabolites in (a) *B. pseudotriquetrum* in ESI+, (b) *B. pseudotriquetrum* in ESI‐, (c) *P. patens* in ESI+, and (d) *P. patens* in ESI‐, under cold stress. The *x*‐axis shows the log^2^FC (fold change) and the *y*‐axis represent the –log^10^ of the *p*‐values. The red color represents the down‐regulated significant changes in cold stress (VIP > 1, *p* < .05, FC < 0.5), while the green color represents the up‐regulated significant changes in cold stress (VIP > 1, *p* < .05, FC > 1). The blue color shows the variables of VIP > 1, but *p* > .05. For full annotation please see Figure S3a–d

The potential biomarkers for cold stress were screened based on three criteria (fold change of larger than 2 or smaller than 0.5, *p* < .05, and VIP >1). The lipid molecular species that fulfilled all three criteria were designated as biomarkers for cold stress (Figure 3). Detailed lists for biomarker screening can be found in Data S2 and Figure S3a–d. In *B. pseudotriquetrum*, several membrane lipids (mostly PC, such as PC 34:2, and PC 18:2_20:4, and PG 16:0_16:0, PI 34:2), as well as most of the 34–38 carbons MGDG and DGDG with C16‐18 FAs, showed an increase under cold stress (Figure S4). In ESI‐ mode, all identified PMeOH lipids were up‐regulated under cold stress, together with PE 16:0_18:2, PE 16:0_20:4, and PI 16:0_18:2. The storage lipids, including DG and TG, in contrast, are down‐regulated under cold stress. In *P. patens*, most of the TG lipids decreased under cold stress, but several DG lipids (e.g., DG 16:0_18:1, DG 16:0_20:3, and DG 18:1_18:3) and PG (PG 16:1_18:3 and PG 16:1_18:2) increased (Figure S4).

**FIGURE 4:**
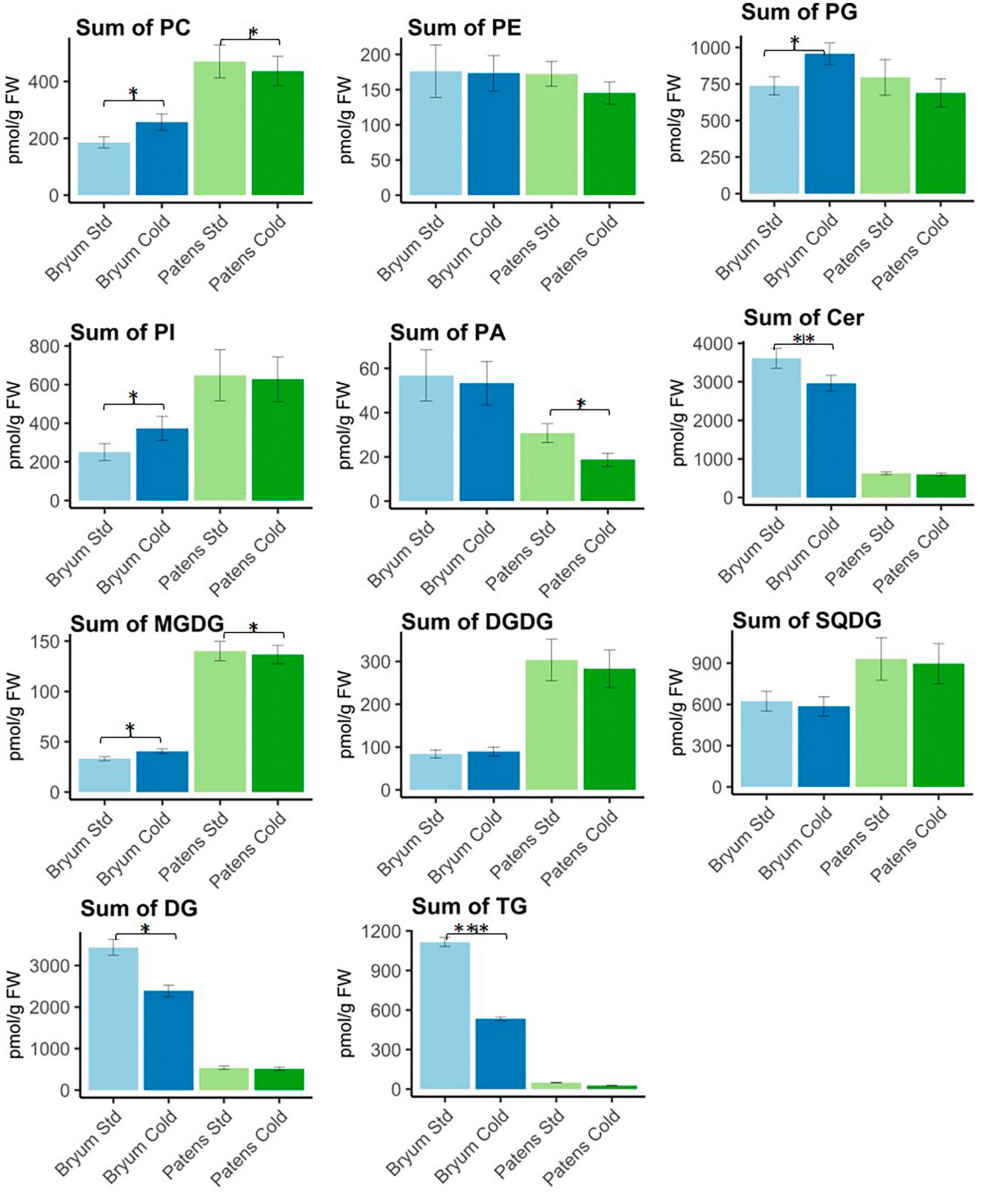
Sums of different lipids classes in *B. pseudotriquetrum* cultivated at 23°C (light blue), *B. pseudotriquetrum* cultivated at 10°C (dark blue), *P. patens* cultivated at 23°C (light green), *P. patens* cultivated at 10°C (dark green). Two‐tailed *t*‐test (*n* = 3) was performed to calculate the significant differences (*p ≤ .05, **p ≤ .01, ***p≤ .001) and error bars indicate the standard deviation. Cer, ceramide; DG, diglyceride; DGDG, digalactosyldiacylglycerol; MGDG, monogalactosyldiacylglycerol; PA, phosphatidic acid; PC, phosphatidylcholine; PE, phosphatidylethanolamine; PG, phosphatidylglycerol; PI, phosphatidylinositol; SQDG, sulfoquinovosyldiacylglycerol; TG, triglyceride; the quantification of lipids was calculated by using internal standards representing each lipid classes with a few exceptions, PA, was normalized by Cer 18:1;2O/16:0(d7), and MGDG, DGDG, and SQDG were normalized by DGTS d9

Four out of five identified PMeOH lipids (PMeOH 16:0_18:1, PMeOH 16:0_18:2, PMeOH 16:0_18:3, and PMeOH 16:0_20:4) showed significant increase in both moss species (Figure S4). The presence of PMeOH is assumed that the choline head group from PC reacts with methanol when phospholipase D is active and generates PMeOH and free choline. However, we speculate the PMeOH is also produced endogenously in *P. patens* since the total amount of PC decreased while total amount of PMeOH increased (Figure 4, Data S3).

## 4 DISCUSSION

The major phospholipids, signaling lipids and storage lipids found in both moss species are common lipid classes also found in algae, higher plants, and other bryophytes (Chen et al., 2013; Conde et al., 2021; Okazaki & Saito, 2018; Vu et al., 2014), and the change observed here (Figure 4) is not unusual, though some for plants unusual lipids was found to be biosynthesized in bryophytes.

The unusual lipid class PMeOH detected in both moss species has been reported to be an artifact from lipid extraction when using methanol (Roughan et al., 1978). The PMeOH lipid was identified by its characteristic m/z 110.981 (CH4OP‐) in ESI‐ mode, which corresponds to the head group of a phosphatidic acid with a methyl group added to the phosphate, an example of the MS/MS spectrum of PMeOH 16:0_18:2 is shown in Figure S5. Tsugawa et al. (2019) analyzed the lipid compositions of nine algal species, but only detected several PMeOH lipids in one of them, *Euglena gracilis*, even if all algal species were extracted with the same method. In our study, the moss materials were kept at −20°C right after harvest, and were ground in liquid nitrogen to quench the lipid metabolism, before lipid extraction to limit methylation. A difference in the cold response is also seen for PC and PMeOH, for example, PC 16:0_18:2 decreased in cold stress in *P. patens*, whereas PMeOH 16:0_18:2 increased (Data S3). The different cold response of PC and PMeOH suggests that PMeOH is likely arise from a biosynthetic pathway in both mosses.

Another unusual lipid class, sulfonolipids (SLs, or *N*‐acyl‐capnine) again detected in both moss species, are structurally related to ceramides, but has a sulfonic acid group in the sphingoid base (Walker et al., 2017). Five SLs and oxidized SLs (O‐acetylated SL) were identified as [M‐H]‐ adducts by their characteristic m/z 79.958, which represents the sulfite group, and a neutral loss of the fatty acyl from the sphingoid base, an example of MS/MS spectrum of SL (17:0;0/17:1) is shown in Figure S5. SLs were previously only described in diatoms (Anderson et al., 1978) and some bacterial species (Walker et al., 2017). SL is likely produced by N‐acylation of its precursor capnine with fatty acids (Godchaux & Leadbetter, 1980). The presence of SLs may again suggest that bryophytes have different biosynthetic pathways for lipids than vascular plants. Alternatively, the SL could arise from a bacterial contamination in the liquid culture though we did not observe this in our previous study by using nonaxenic moss materials (Lu et al., 2021).

Among the more common lipids, considerable amounts of vl‐PUFAs were detected in both moss species, including FA's longer than 20 carbons as seen in previous studies (Beike et al., 2014; Girke et al., 1998), that also described 24:0, 25:0, and 26:0 FAs in several moss species. It is known that vl‐PUFAs provide freezing tolerance for the mosses (Glime, 2017; Hansen & Rossi, 1991; Hartmann et al., 1986; Lu et al., 2019), one may expect to find higher PUFAs in association with phospholipids and glycolipids. However, the level of total amount of FA in *B. pseudotriquetrum* did not change under cold stress, which would have been expected, and for PG 32:0(16:0_16:0) and DGDG 32:0 (16:0_16:0) these increased under cold stress, whereas DGDG 40:8 (20:4_20:4) decreased. A recent study investigated the lipid changes of wild type *P. patens* under cold stress (Resemann, 2018), the author found that polyunsaturated C16 and C18 FAs decreased, but vl‐PUFAs (C20 and above) accumulated in phospholipids and glycolipids. The results here show that the 20:4 and 20:5 containing lipids accumulated mostly in PC, MGDG and DG (Figure 3, Data S3). The biosynthesis and the response to cold stress is not the same in the two species. The data suggest that for *B. pseudotriquetrum* the biosynthesis of vl‐PUFA might not be regulated by temperature but be a consistent production that ensure a constant resistant to temperature changes, whereas in *P. patens* there is a clear response in the overall level.

*B. pseudotriquetrum* and *P. patens* have a similar lipid composition in terms of the lipid molecular species, but their individual lipid classes vary substantially in a quantitative level (Figure 4). Similar quantities of phospholipids were observed for some, but *B. pseudotriquetrum* has significant higher amounts of storage lipids (DG and TG), signaling lipid Cer and FA. In contrast, *P. patens* has higher amounts of glycolipids (MGDG, DGDG, and SQDG). The high concentration of glycolipids in *P. patens* are mostly contributed by C16‐18 carbon lipids such as MGDG 16:2_18:2, MGDG 16:2_18:3, MGDG 16:3_18:3, DGDG 16:0_18:2, and SQDG 16:0_18:2. *B. pseudotriquetrum*, on the other hands, contains high amounts of 20:4‐containing lipids such as DG 16:0_20:4, DG 20:4_20:4, TG 18:3_20:4_20:4, TG 20:4_20:4_20:4, and FA 20:4.

In *B. pseudotriquetrum*, accumulation of PC with at least one polyunsaturated fatty acyl chain, such as PC 34:2 (16:0_18:2), PC 34:3 (16:0_18:3), PC 36:4 (16:0_20:4), and PC 38:7 (18:3_20:4), during cold stress, was observed. In contrast, lipid species with two saturated or mono‐saturated fatty acyl chains, such as PC 32:0 (16:0_16:0), and PC 32:1 (16:0_16:1) decreased (Data S3). Similar to PC, MGDG with higher degrees of unsaturation levels, such as MGDG 36:6 (18:3_18:3), MGDG 34:6 (16:3_18:3), and MGDG 38:6 (18:2_20:4), increased under cold stress, while MGDG 34:1 (16:0_18:1) decreased. Those findings are generally consistent with previous studies of higher plants or algae and show that this might an early evolutionary adaption to cold temperatures (Chen et al., 2013; Gao et al., 2019; Resemann, 2018; Wang et al., 2006).

For other lipids classes, such as PG, the data showed that this is the only lipid class in which no FA 20:4 and above were observed in both moss species. This result matches with Resemann, 2018, who studied the lipid composition of *P. patens*. However, the reason why PG lipid class contains no FA 20:4 remains unclear. For TG the total content decreased significantly in both *B. pseudotriquetrum* and *P. patens* (Figure 4), which indicates the TG breaks down in the moss cells when exposed to cold stress (Chen et al., 2013), and use the FAs released from TG for synthesis of phospholipids and glycolipids. Resemann (2018) reported a slight increase of TG in wild type *P. patens* under cold stress at 4°C. However, they have conducted a much longer stress period (7 days), we therefore assume the TG synthesis undergoes a decrease when exposed to acute cold stress (24 h), then recovers gradually afterwards. The regulation of TG in plants is not yet well‐studied, possibly due to the fact that TG lipids have complex structures and several combinations of fatty acyls, which may cause false identifications based on their MS/MS spectrums (Tsugawa et al., 2019).

## 5 CONCLUSION

Using mass spectrometry‐based lipidomic approach, we identified hundreds of lipids with high quality MS/MS spectrums in moss *B. pseudotriquetrum* and *P. patens*. The lipidomes of *Bryum pseudotriquetrum* is reported the first time. Unusual lipids such as sulfonolipids (SL), phosphatidylmethanol (PMeOH), and hydrogenated glycolipid DGDG 32:0, were found for the first time in both moss species and provide indications that these lipids are biosynthesized in the moss cells. *B. pseudotriquetrum* and *P. patens* showed clearly different responses to cold stress. We confirmed 25 and 26 lipids were found to be up‐regulated and 43 and 69 lipids were downregulated during cold stress in *B. pseudotriquetrum* and *P. patens*, respectively. Overall, this work provides further insight of the cold stress adaptation in bryophytes with specific focus on the lipid metabolism, and show that the various lipid families are metabolized differently between bryophytes to react to cold stress.

## CONFLICT OF INTEREST

The authors declare no conflict of interest.

## Supporting information


Data S1

Data S2

Data S3

Appendix S1

## Data Availability

All data that support the findings of this study are available at DTU data repository, Lu et al (2022): Cold stress paper. Technical University of Denmark. Dataset. https://doi.org/10.1002/pei3.10095.
