# Supplementary figures and images for: Lipidomic analysis of moss species Bryum pseudotriquetrum and Physcomitrium patens under cold stress
Source: Plant Environ Interact. 2022 Dec 22;3(6):254–63. doi: 10.1002/pei3.10095 (PMC10168071; doi:10.1002/pei3.10095)

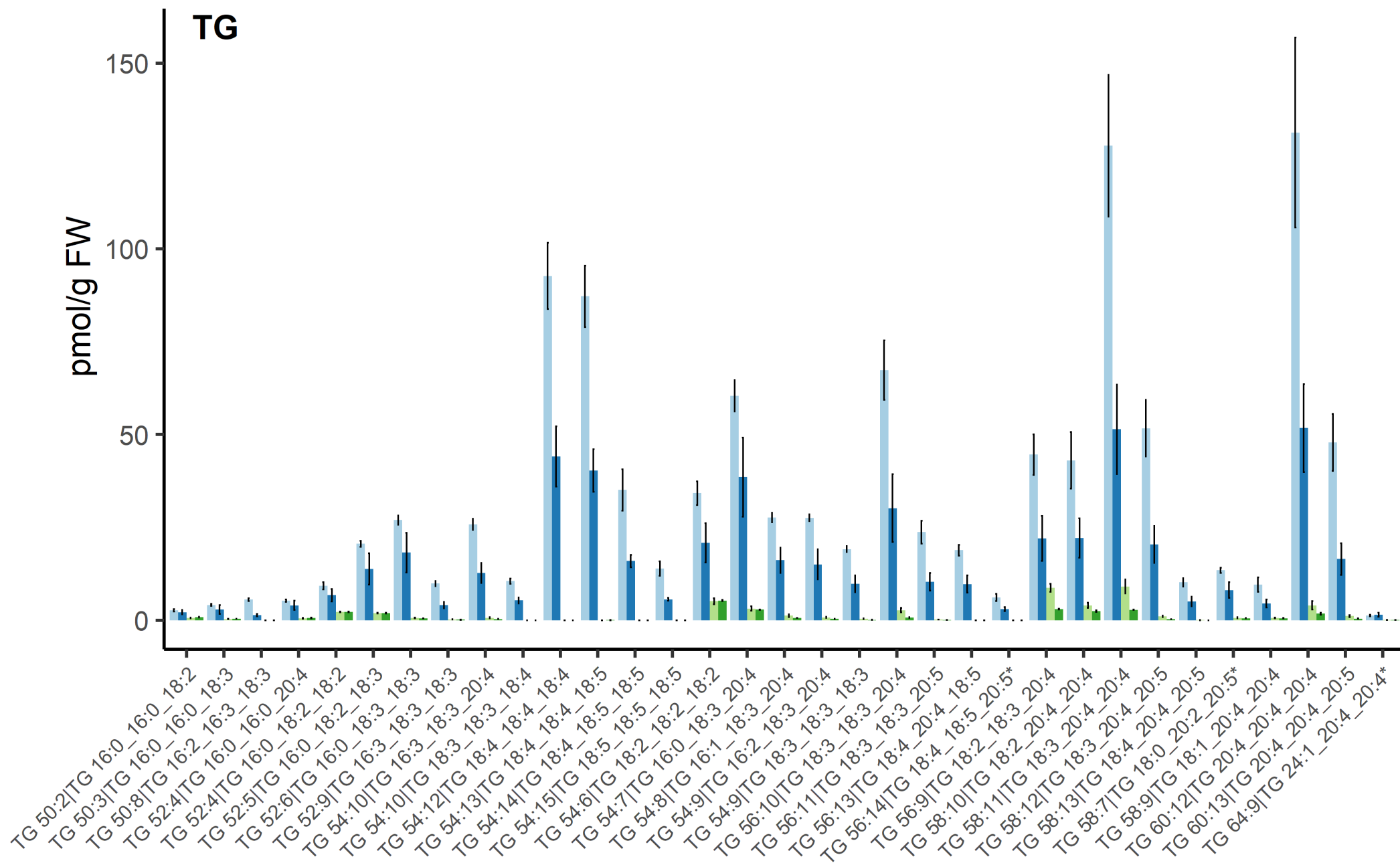

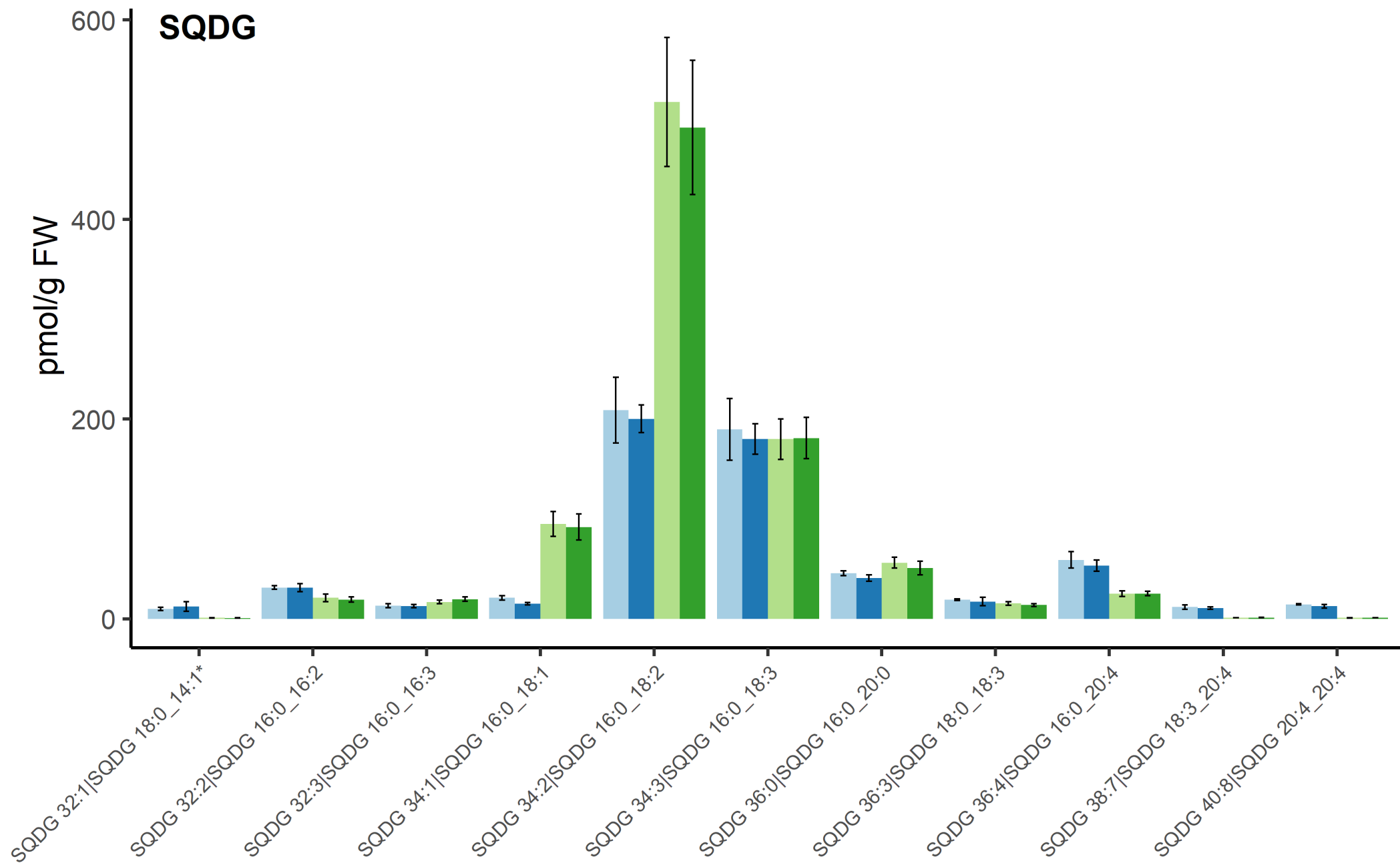

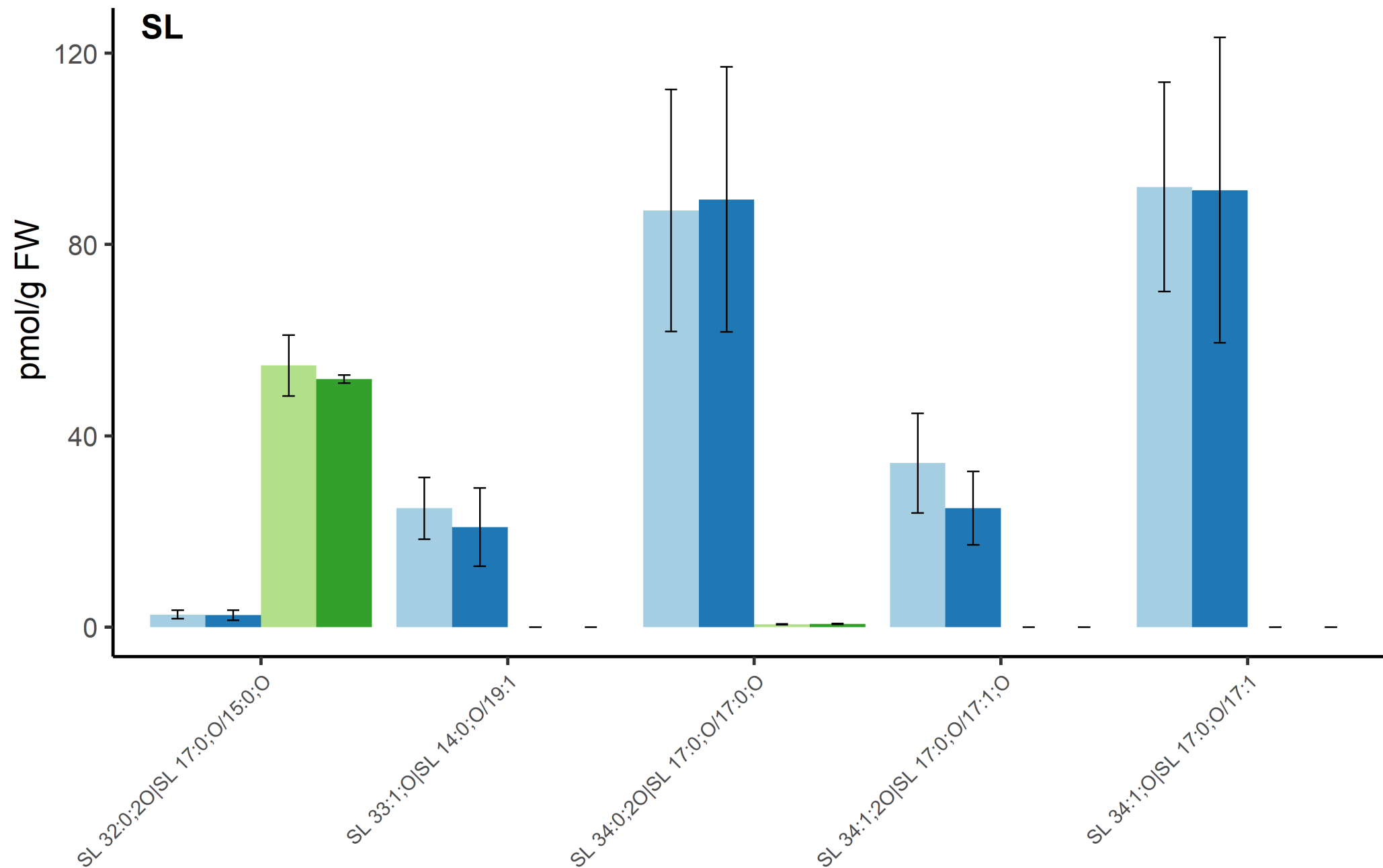

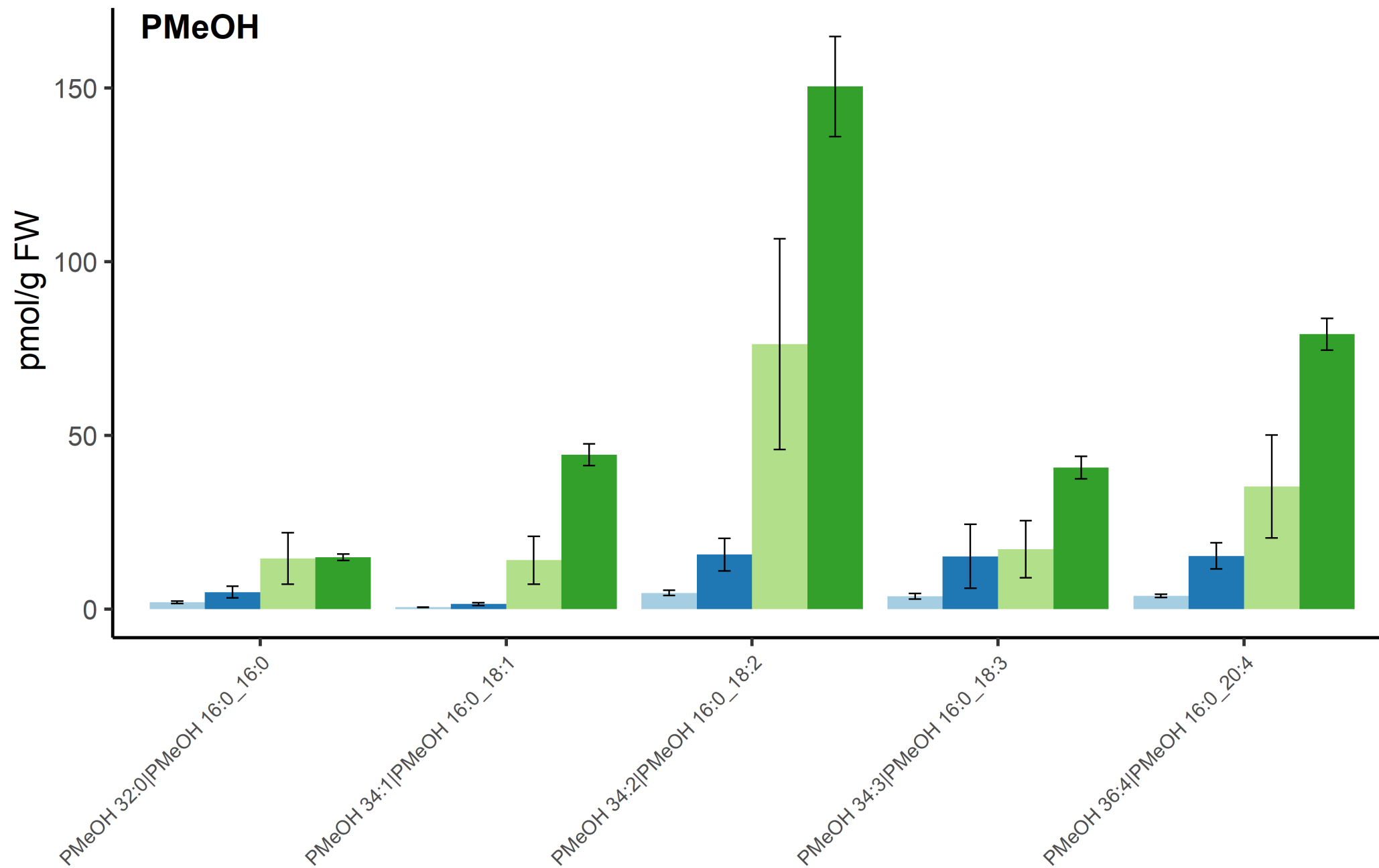

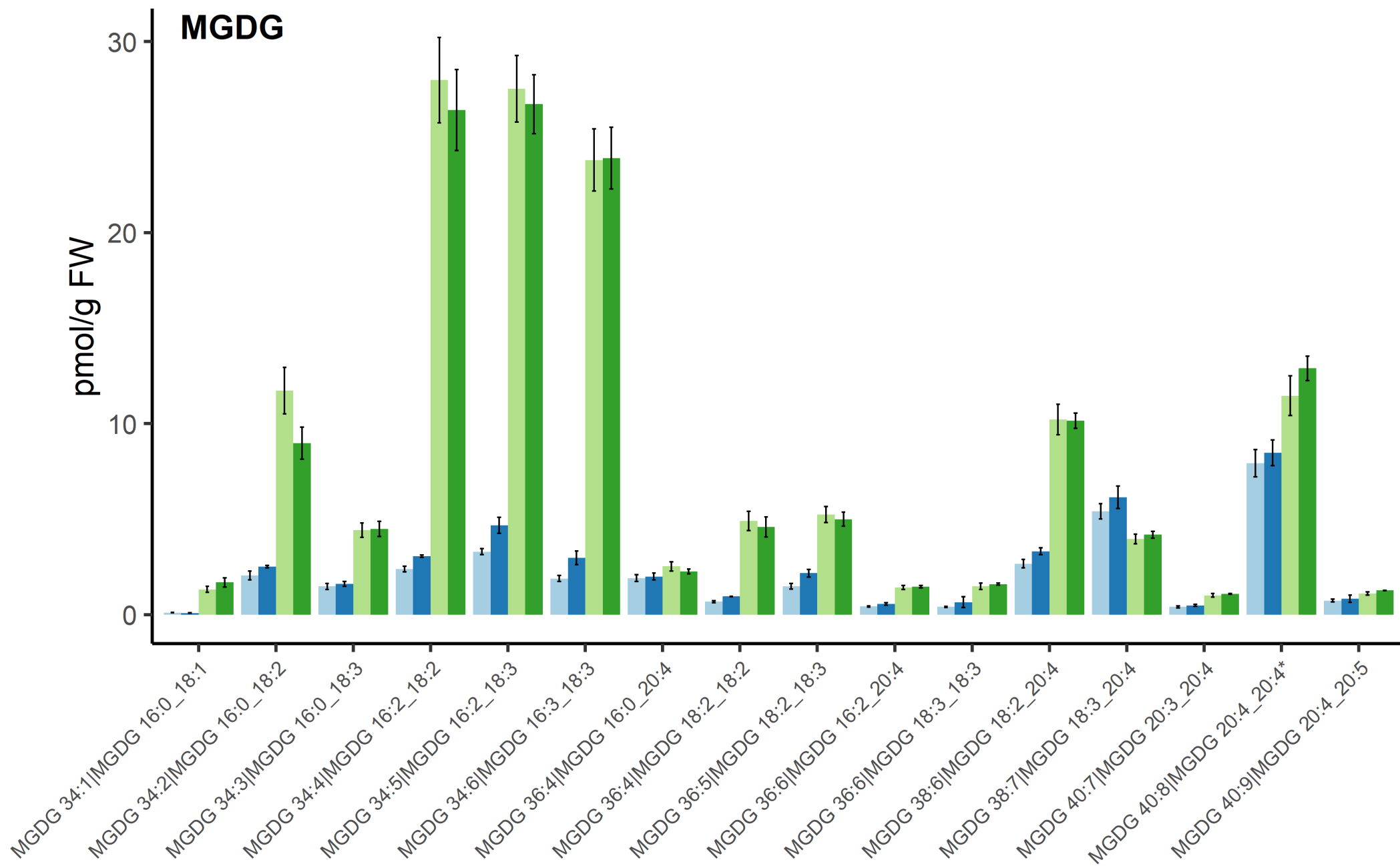

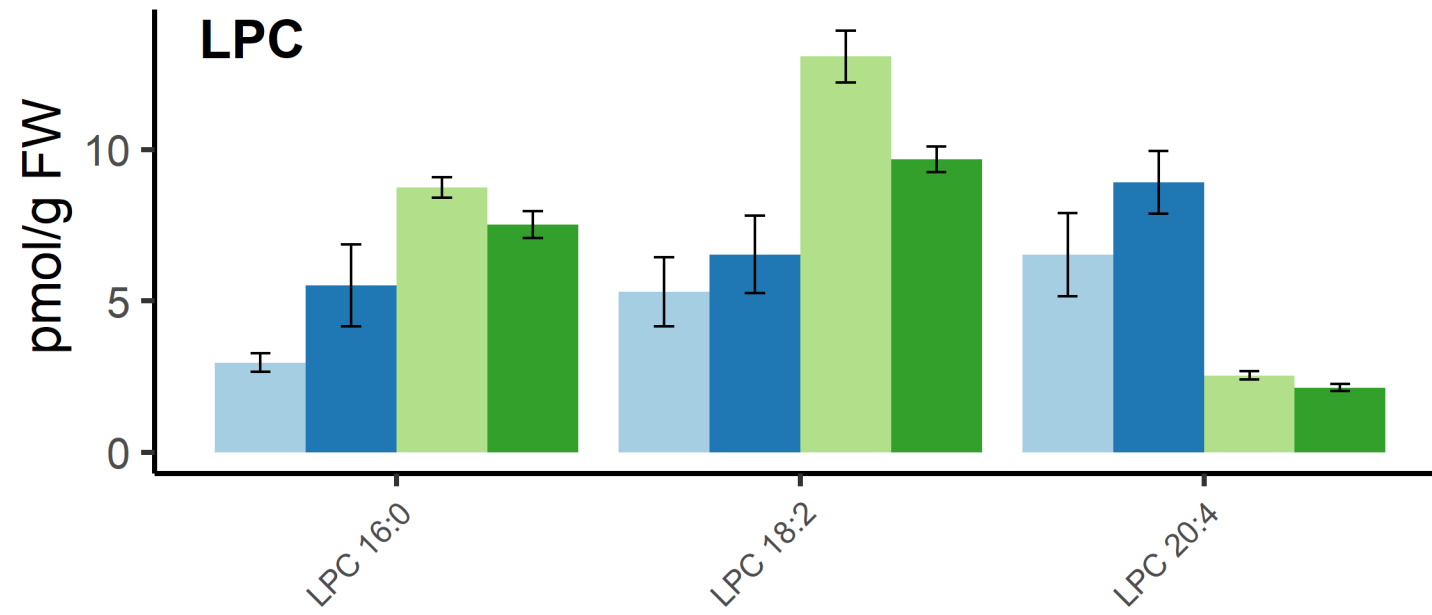

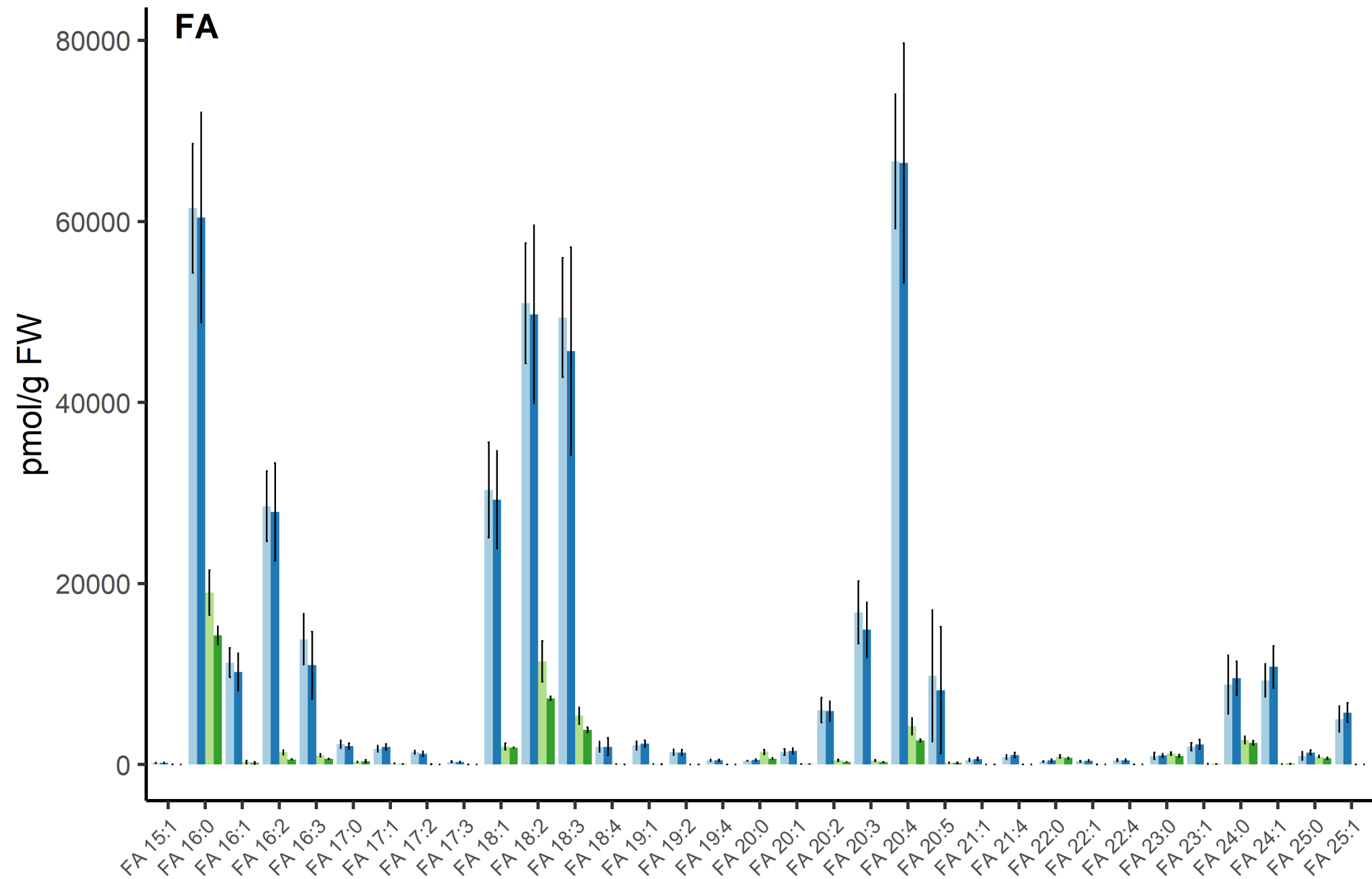

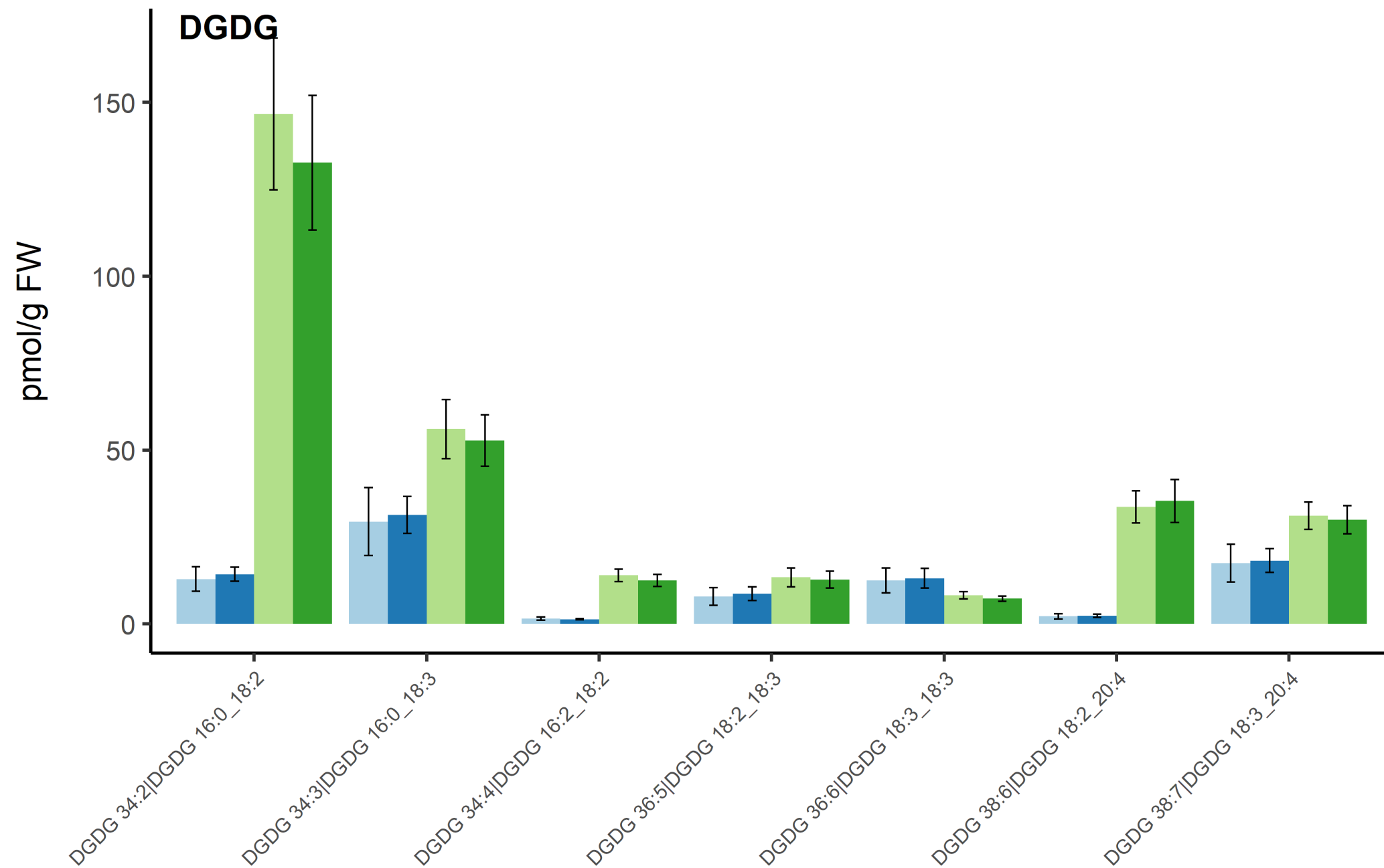

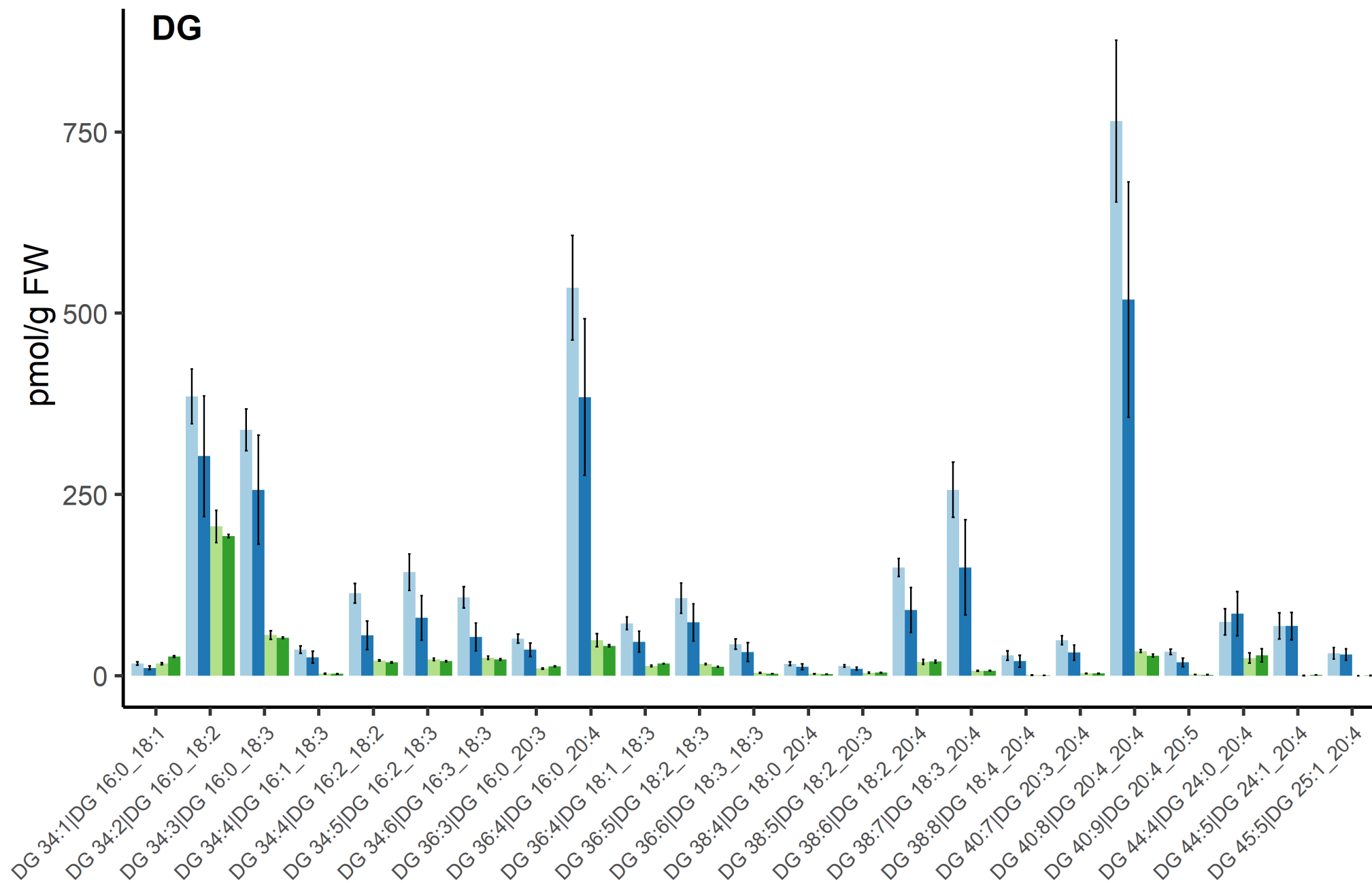

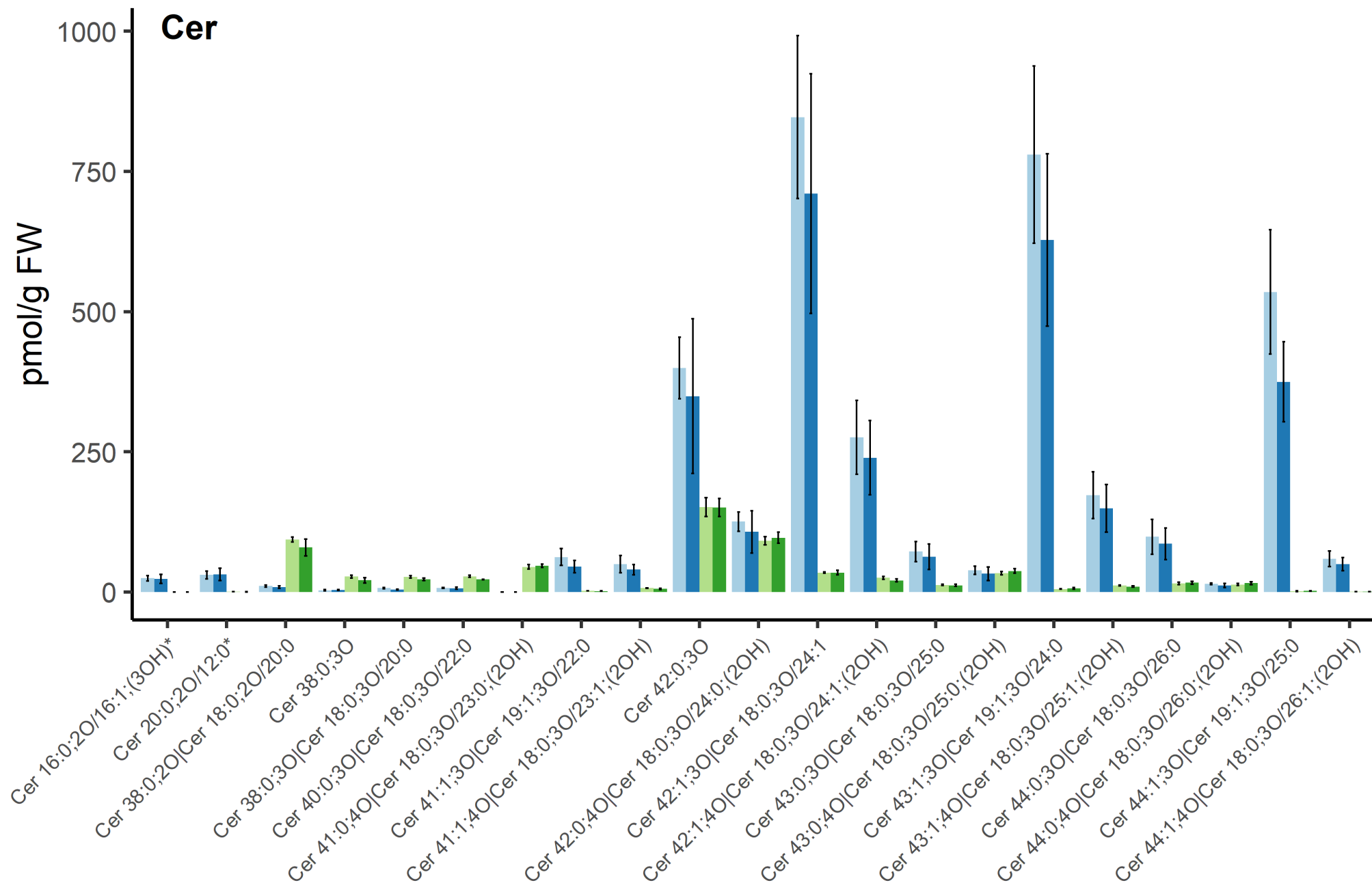

Supplement: Supplementary file 3 — Data S3 [file PEI3-3-254-s004.pdf]
