# Supplementary material for: Lipidomic analysis of moss species Bryum pseudotriquetrum and Physcomitrium patens under cold stress
Source: Plant Environ Interact. 2022 Dec 22;3(6):254–63. doi: 10.1002/pei3.10095 (PMC10168071; doi:10.1002/pei3.10095)
Supplement: Supplementary file 4 — Appendix S1 [file PEI3-3-254-s001.pdf]

Supplementary material

Lipidomic analysis of moss species *Bryum pseudotriquetrum* and *Physcomitrium patens* under cold stress

Yi Lu[1,2], Finnur Freyr Eiriksson[2,3], Margrét Thorsteinsdóttir[2,3] and Henrik Toft Simonsen[1,*]

1. Department of Biotechnology and Biomedicine, Technical University of Denmark, Søltofts Plads 223, 2800 Kongens Lyngby, Denmark; yilu@dtu.dk (Y.L), hets@dtu.dk (H.T.S)
2. ArcticMass, Sturlugata 8, 101 Reykjavik, Iceland; finnur@arcticmass.is (F.F.E); margreth@hi.is (M.T)
3. Faculty of Pharmaceutical Sciences, University of Iceland, Hagi, Hofsvallagata 53, 107 Reykjavik, Iceland

Table and figure legends

Supplementary Figure S1. Recovery of internal standard used for calculation of lipid relative concentration. PC, phosphatidylcholine; LPC, lysophosphatidylcholine; CE, cholesterol ester; MG, monodiacylglyceride; DG, diglyceride; TG, triglyceride; SM, sphingomyelin; Cer, ceramides; DGTS, diacylglyceryl-N,N,N-trimethylhomoserine; PE, phosphatidylethanolamine; PS, phosphatidylserine; PG, phosphatidylglycerol; PI, phosphatidylinositol; LPE, lysophosphatidylethanolamine.

Supplementary Figure S2. PCA plot of dataset in ESI+ mode (R2X = 0.881, Q2X= 0.856) and ESI- mode (R2X = 0.984, Q2X = 0.976). Hotelling´s T2 = 95%. *B. pseudotriquetrum* samples are marked in green and P. patens are marked in blue. QC (red color) represents quality control samples.

Supplementary Figure S3. Lipid quantification of individual lipid species in *B. pseudotriquetrum* cultivated at 25 °C (light blue), *B. pseudotriquetrum* cultivated at 10 °C (dark blue), *P. patens* cultivated at 25 °C (light green), and *P. patens* cultivated at 10 °C (dark green). Error bars indicate the standard deviation of three replicates. PC, phosphatidylcholine; PE, phosphatidylethanolamine; PG, phosphatidylglycerol; PI, phosphatidylinositol; PA, phosphatidic acid; MGDG, monogalactosyldiacylglycerol; DGDG, digalactosyldiacylglycerol; SQDG, sulfoquinovosyldiacylglycerol. DG, diglyceride; TG, triglyceride; Cer, ceramides; PMeOH, phosphatidylmethanol; LPC, lysophosphatidylcholine; SL, sulfonolipid. The quantification of lipids were calculated by using internal standards representing each lipid classes with a few exceptions, PA, PMeOH, and SL were normalized by Cer 18:1;2O/16:0(d7) , and MGDG, DGDG, and SQDG were normalized by DGTS d9. Lipid species marked with "*" are tentative identification without full match of MS2 spectrum (i.e. no fatty acyl chain information).

Supplementary Figure S4. Permutation tests of 999 permutations of (A). *B. pseudotriquetrum* in ESI+, (B). *P. patens* in ESI+, (C). *B. pseudotriquetrum* in ESI-, and (D) *P. patens* in ESI-.

Supplementary Figure S5. Examples of MS/MS spectrums of unusual lipid detected in mosses, A). Phosphatidylmethanol (PMeOH 16:0_18:2). B). Sulfnolipids (SL 17:0;O/17:1). NL, neutral loss.

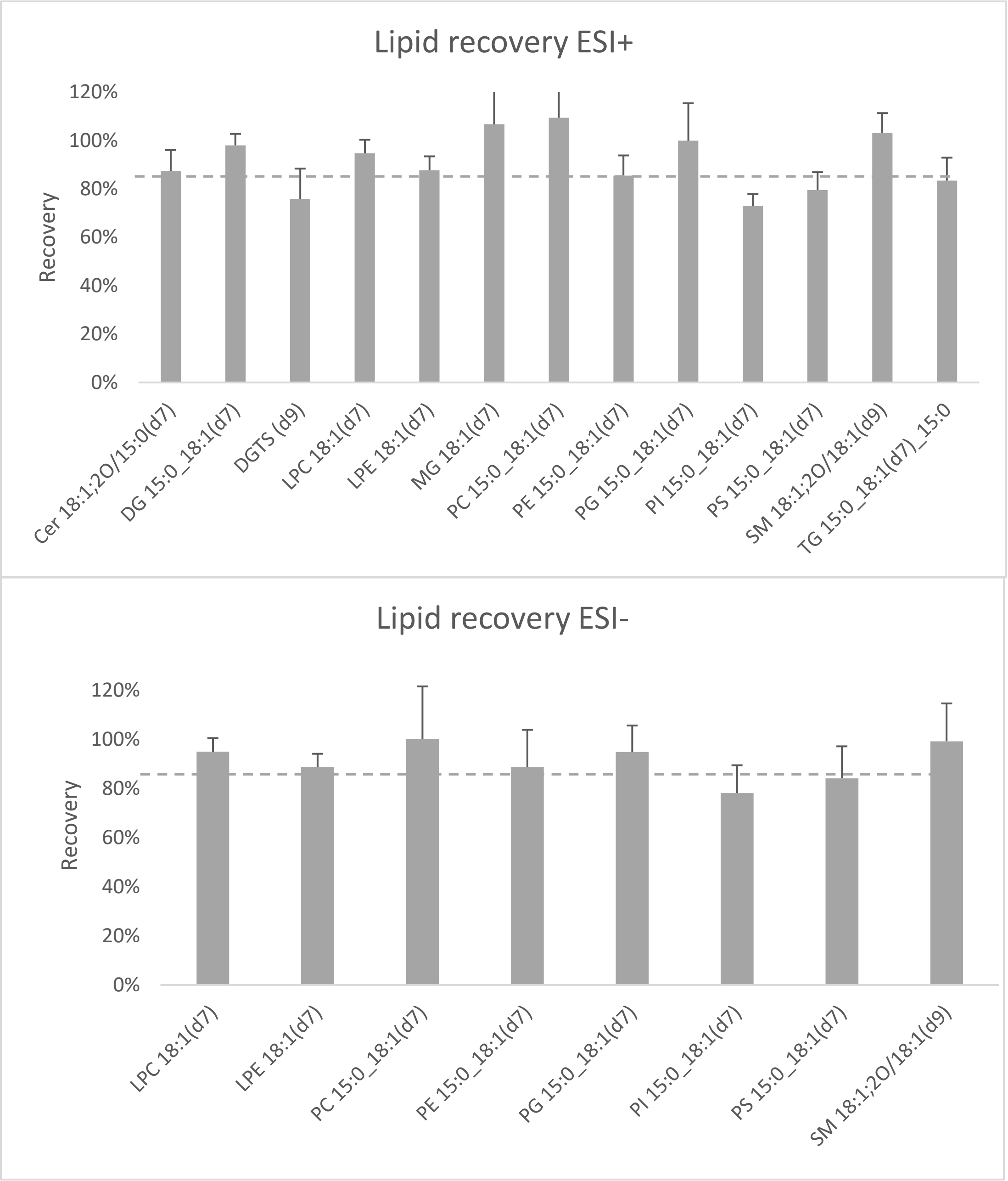

Supplementary Figure S1. Recovery of internal standard used for calculation of lipid relative concentration. PC, phosphatidylcholine; LPC, lysophosphatidylcholine; CE, cholesterol ester; MG, monodiacylglyceride; DG, diglyceride; TG, triglyceride; SM, sphingomyelin; Cer, ceramide; DGTS, diacylglyceryl-N,N,N-trimethylhomoserine; PE, phosphatidylethanolamine; PS, phosphatidylserine; PG, phosphatidylglycerol; PI, phosphatidylinositol; LPE, lysophosphatidylethanolamine.

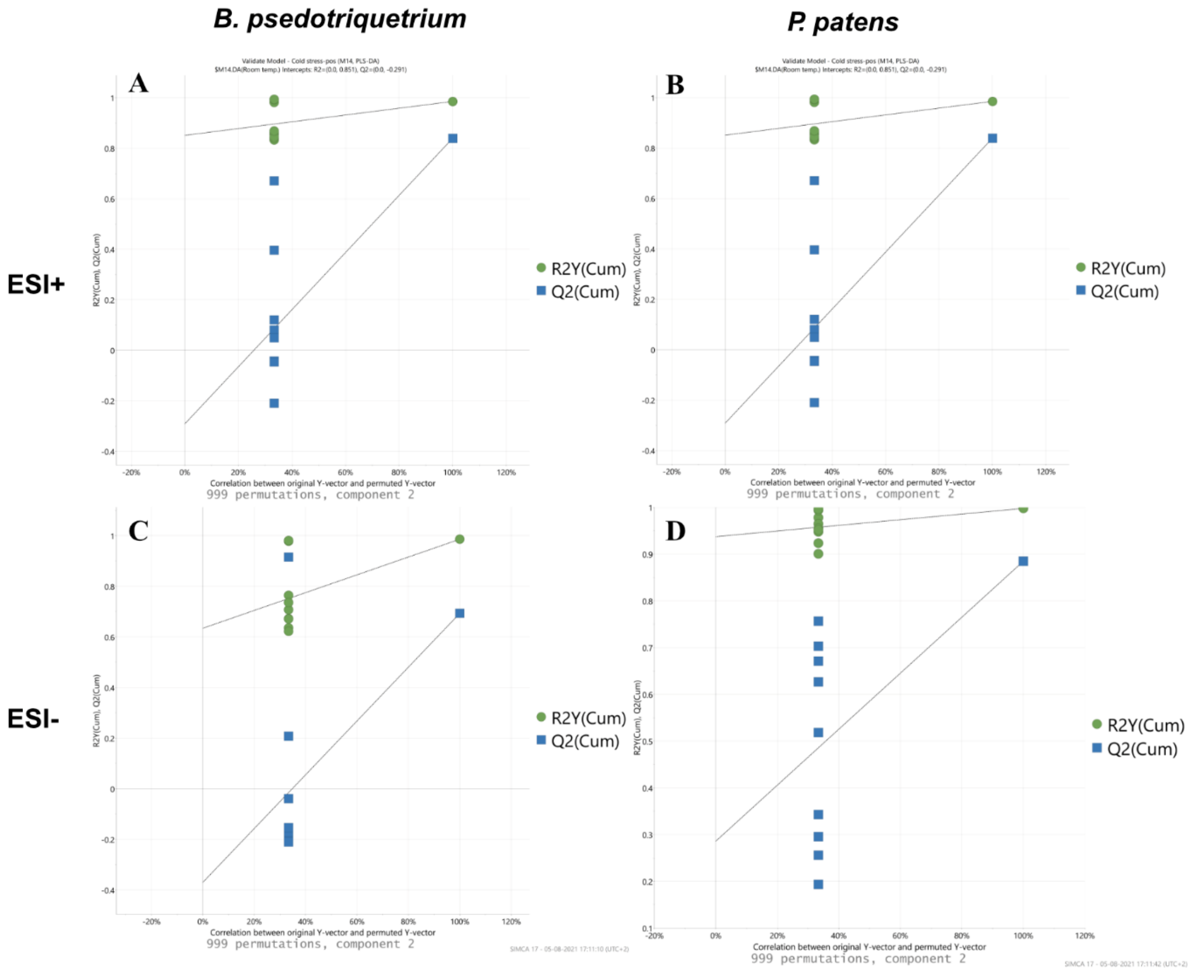

Supplementary Figure S2. Permutation tests of 999 permutations of (A). *B. pseudotriquetrum* in ESI+, (B). *P. patens* in ESI+, (C). *B. pseudotriquetrum* in ESI-, and (D). *P. patens* in ESI-.

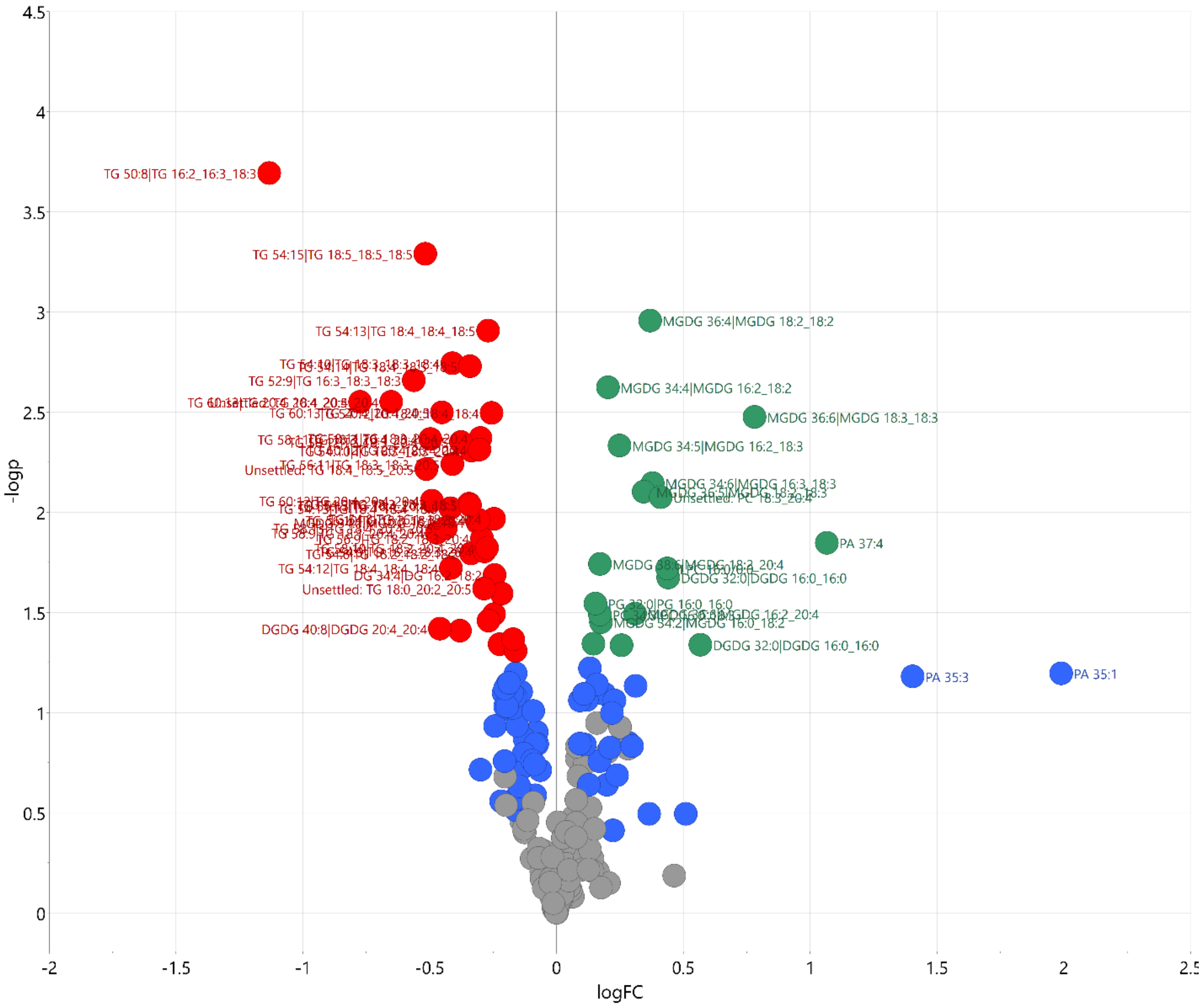

Supplementary Figure S3A. Volcano plots of significant changes of identified lipid metabolites in *B. pseudotriquetrum* in ESI+. The x-axis shows the log^{2}FC (fold change) and the y-axis represent the –log^{10} of the p-values. The red color represents the down-regulated significant changes in cold stress (VIP>1, p<0.05, FC<0.5), while the green color represents the up-regulated significant changes in cold stress (VIP>1, p<0.05, FC>1). The blue color shows the variables of VIP>1, but p>0.05.

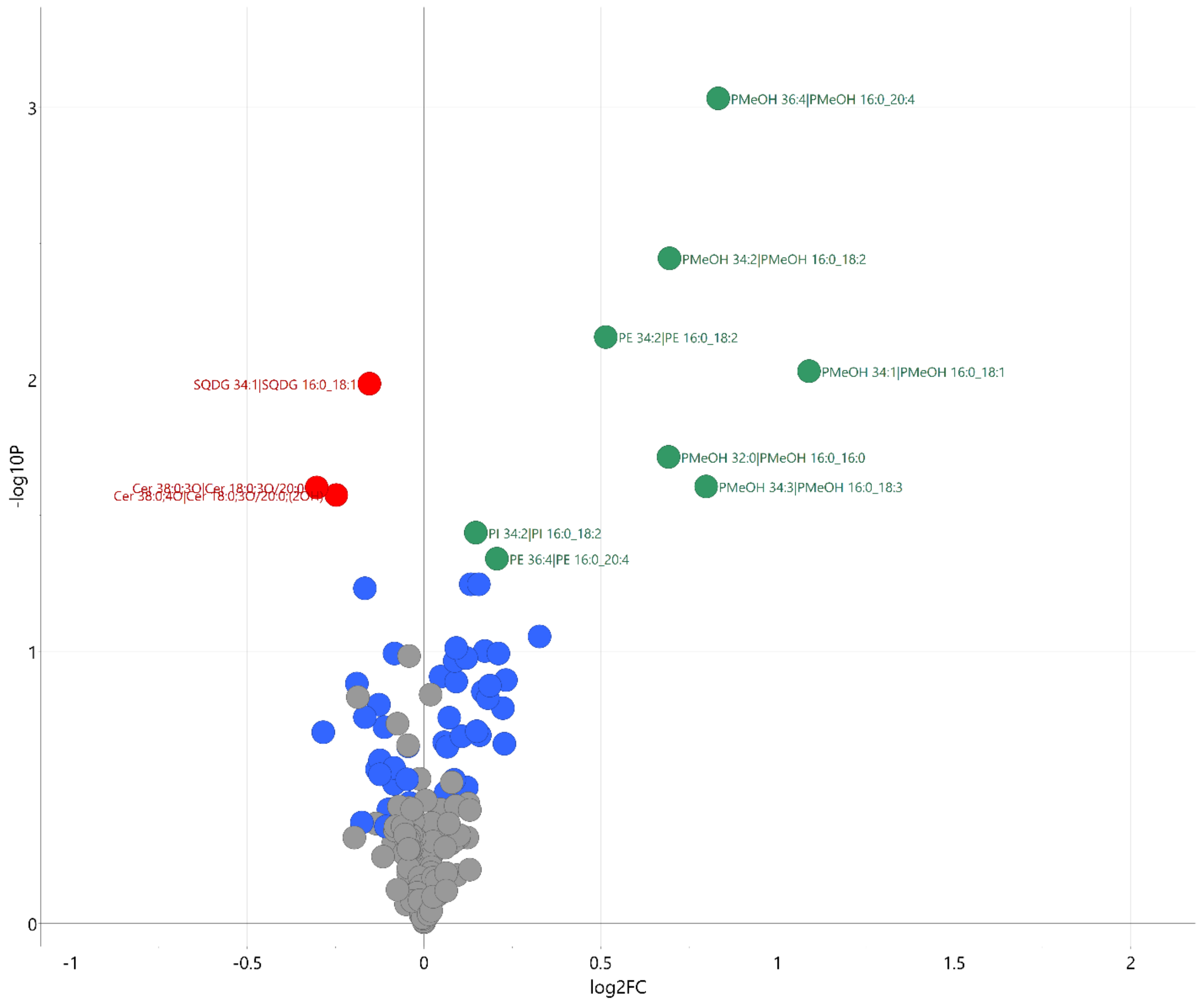

Supplementary Figure S3B. Volcano plots of significant changes of identified lipid metabolites in *B. pseudotriquetrum* in ESI- under cold stress. The x-axis shows the log^2FC (fold change) and the y-axis represent the $–log^{10}$ of the p-values. The red color represents the down-regulated significant changes in cold stress (VIP>1, p<0.05, FC<0.5), while the green color represents the up-regulated significant changes in cold stress (VIP>1, p<0.05, FC>1). The blue color shows the variables of VIP>1, but p>0.05.

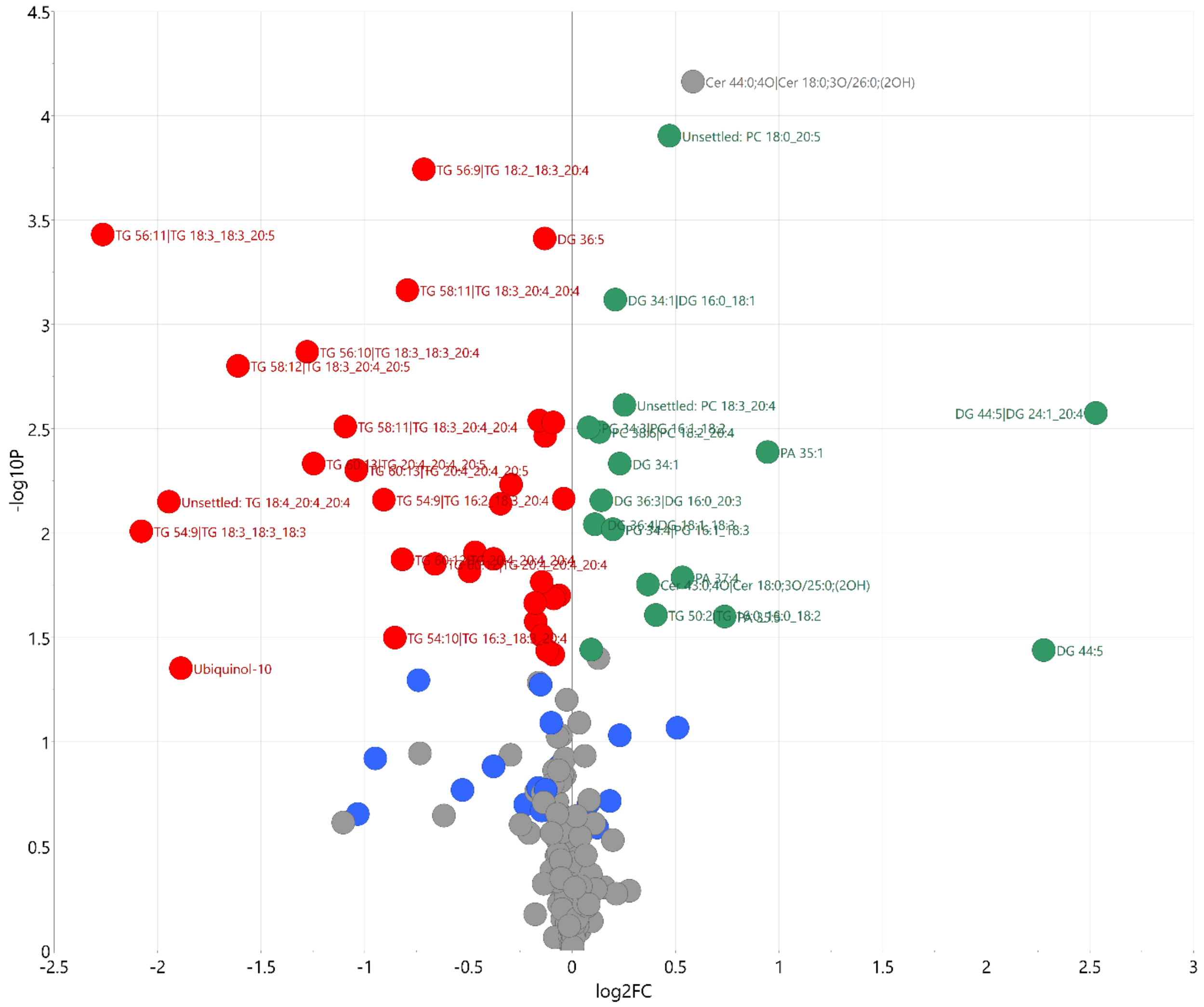

Supplementary Figure 3C. Volcano plots of significant changes of identified lipid metabolites in *P. patens* in ESI+ under cold stress. The x-axis shows the log^{2}FC (fold change) and the y-axis represent the –log^{10} of the p-values. The red color represents the down-regulated significant changes in cold stress (VIP>1, p<0.05, FC<0.5), while the green color represents the up-regulated significant changes in cold stress (VIP>1, p<0.05, FC>1). The blue color shows the variables of VIP>1, but p>0.05.

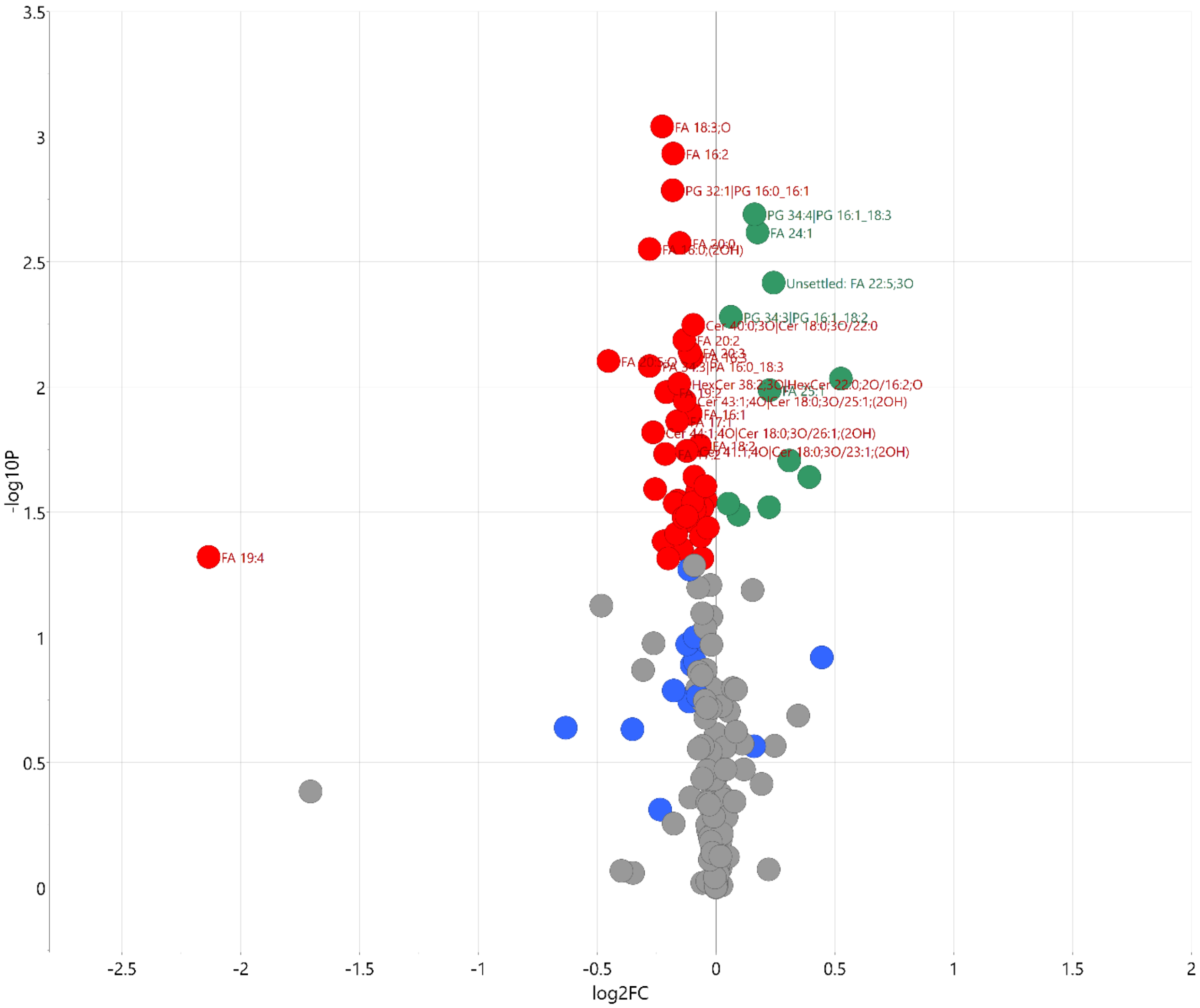

Supplementary Figure 3D. Volcano plots of significant changes of identified lipid metabolites in *P. patens* in ESI- under cold stress. The x-axis shows the log^{2}FC (fold change) and the y-axis represent the –log^{10} of the p-values. The red color represents the down-regulated significant changes in cold stress (VIP>1, p<0.05, FC<0.5), while the green color represents the up-regulated significant changes in cold stress (VIP>1, p<0.05, FC>1). The blue color shows the variables of VIP>1, but p>0.05.

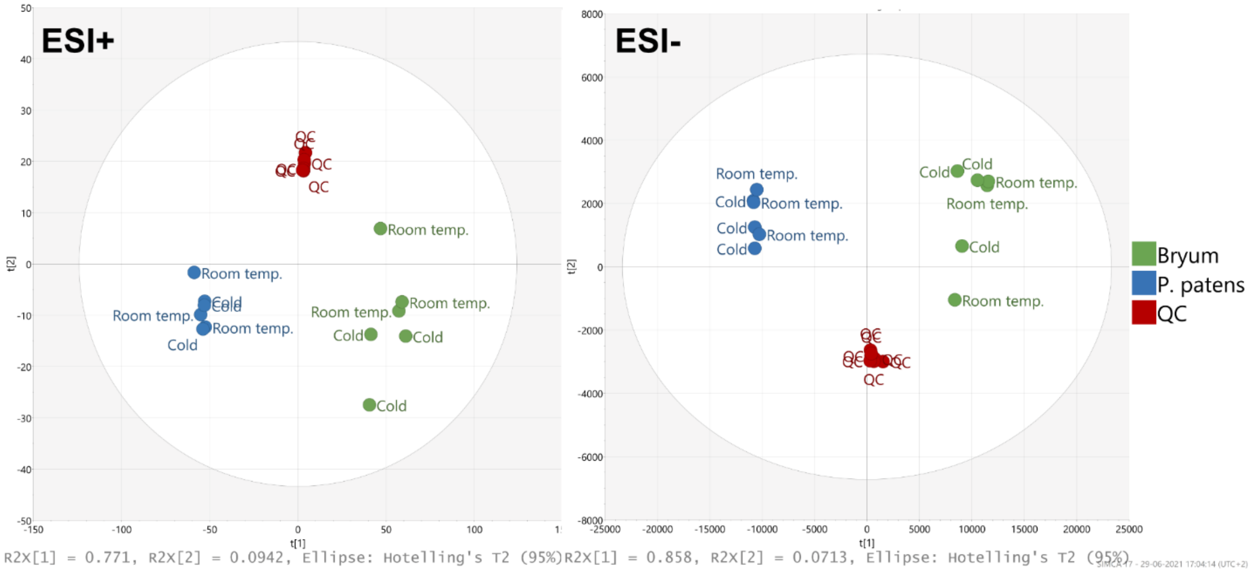

Supplementary Figure S4. PCA plot of dataset in ESI+ mode (R2X = 0.881, Q2X= 0.856) and ESI- mode (R2X = 0.984, Q2X = 0.976). Hotelling´s T2 = 95%. *B. pseudotriquetrum* samples are marked in green and *P. patens* are marked in blue. QC (red color) represents quality control samples.

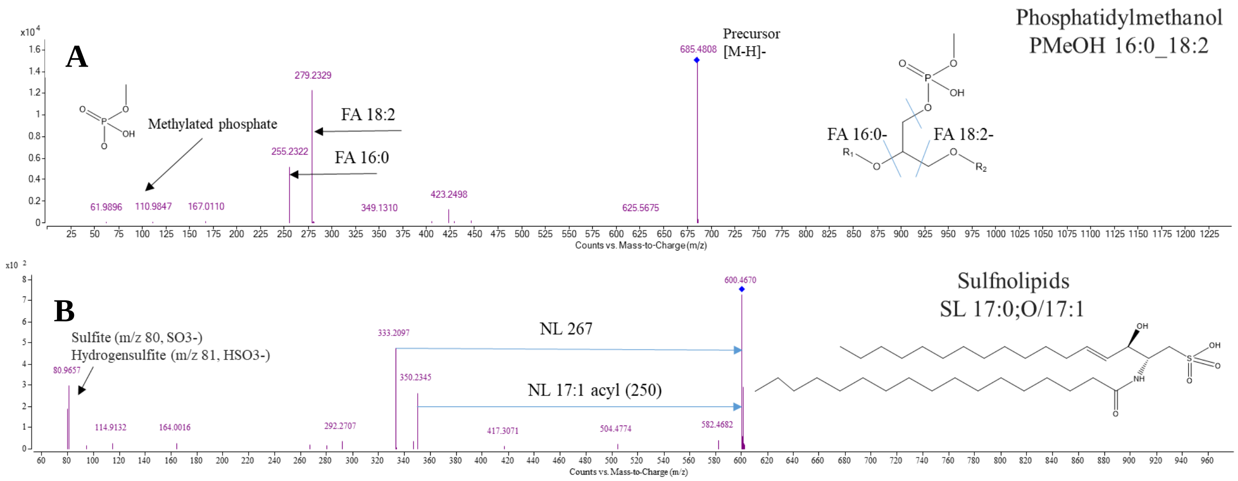

Supplementary Figure S5. Examples of MS/MS spectrums of unusual lipid detected in mosses, A). Phosphatidylmethanol (PMeOH 16:0_18:2). B). Sulfnolipids (SL 17:0;O/17:1). NL, neutral loss.

Supplementary Data S1. List of relative concentrations of identified lipids.

Supplementary Data S2. List of pareto-scaled and log2-tranformed data for biomarker screening and for generating the volcano plots.

Supplementary Data S3. Lipid quantification of individual lipid species in *B. pseudotriquetrum* cultivated at 25 ℃ (light blue), *B. pseudotriquetrum* cultivated at 10 ℃ (dark blue), *P. patens* cultivated at 25 ℃ (light green), and *P. patens* cultivated at 10 ℃ (dark green). Error bars indicate the standard deviation of three replicates.
